# Biallelic *MED27* variants lead to variable ponto-cerebello-lental degeneration with movement disorders

**DOI:** 10.1093/brain/awad257

**Published:** 2023-07-30

**Authors:** Reza Maroofian, Rauan Kaiyrzhanov, Elisa Cali, Mina Zamani, Maha S Zaki, Matteo Ferla, Domenico Tortora, Saeid Sadeghian, Saadia Maryam Saadi, Uzma Abdullah, Ehsan Ghayoor Karimiani, Stephanie Efthymiou, Gözde Yeşil, Shahryar Alavi, Aisha M Al Shamsi, Homa Tajsharghi, Mohamed S Abdel-Hamid, Nebal Waill Saadi, Fuad Al Mutairi, Lama Alabdi, Christian Beetz, Zafar Ali, Mehran Beiraghi Toosi, Sabine Rudnik-Schöneborn, Meisam Babaei, Pirjo Isohanni, Jameel Muhammad, Sheraz Khan, Maha Al Shalan, Scott E Hickey, Daphna Marom, Emil Elhanan, Manju A Kurian, Dana Marafi, Alihossein Saberi, Mohammad Hamid, Robert Spaull, Linyan Meng, Seema Lalani, Shazia Maqbool, Fatima Rahman, Jürgen Seeger, Timothy Blake Palculict, Tracy Lau, David Murphy, Niccolo Emanuele Mencacci, Katharina Steindl, Anais Begemann, Anita Rauch, Sinan Akbas, Ayça Dilruba Aslanger, Vincenzo Salpietro, Hammad Yousaf, Shay Ben-Shachar, Katarina Ejeskär, Aida I Al Aqeel, Frances A High, Amy E Armstrong-Javors, Seyed Mohammadsaleh Zahraei, Tahereh Seifi, Jawaher Zeighami, Gholamreza Shariati, Alireza Sedaghat, Samaneh Noroozi Asl, Mohmmad Shahrooei, Giovanni Zifarelli, Lydie Burglen, Claudia Ravelli, Johannes Zschocke, Ulrich A Schatz, Maryam Ghavideldarestani, Walaa A Kamel, Hilde Van Esch, Annette Hackenberg, Jenny C Taylor, Lihadh Al-Gazali, Peter Bauer, Joseph J Gleeson, Fowzan Sami Alkuraya, James R Lupski, Hamid Galehdari, Reza Azizimalamiri, Wendy K Chung, Shahid Mahmood Baig, Henry Houlden, Mariasavina Severino

**Affiliations:** Department of Neuromuscular Diseases, University College London, Queen Square, Institute of Neurology, London WC1N 3BG, UK; Department of Neuromuscular Diseases, University College London, Queen Square, Institute of Neurology, London WC1N 3BG, UK; Department of Neuromuscular Diseases, University College London, Queen Square, Institute of Neurology, London WC1N 3BG, UK; Department of Biology, Faculty of Science, Shahid Chamran University of Ahvaz, Ahvaz, Iran; Narges Medical Genetics and Prenatal Diagnosis Laboratory, Kianpars, Ahvaz, Iran; Ati Mehr Kasra Genetics Institute, Kianpars, Ahvaz, Iran; Clinical Genetics Department, Human Genetics and Genome Research Institute, National Research Centre, Cairo 12622, Egypt; Wellcome Centre for Human Genetics, University of Oxford and Oxford NIHR Biomedical Research Centre, Oxford, OX3 7BN UK; Neuroradiology Unit, IRCCS Istituto Giannina Gaslini, 16147 Genoa, Italy; Department of Pediatric Neurology, Golestan Medical, Educational, and Research Center, Ahvaz Jundishapur University of Medical Sciences, Ahvaz, Iran; Human Molecular Genetics Laboratory, Health Biotechnology Division, National Institute for Biotechnology and Genetic Engineering (NIBGE) College, PIEAS, 44000 Faisalabad, Pakistan; University Institute of Biochemistry and Biotechnology, PMAS Arid Agriculture University, 46300 Rawalpindi, Pakistan; Department of Medical Genetics, Next Generation Genetic Polyclinic, Mashhad, Iran; Molecular and Clinical Sciences Institute, St. George’s, University of London, London SW17 0RE, UK; Innovative Medical Research Center, Mashhad Branch, Islamic Azad University, Mashhad, Iran; Department of Neuromuscular Diseases, University College London, Queen Square, Institute of Neurology, London WC1N 3BG, UK; Department of Medical Genetics, Istanbul Faculty of Medicine, Istanbul University, 34093 Istanbul, Turkey; Department of Neuromuscular Diseases, University College London, Queen Square, Institute of Neurology, London WC1N 3BG, UK; Genetic Division, Pediatrics Department, Tawam Hospital, Al Ain, UAE; School of Health Science, Division Biomedicine and Translational Medicine, University of Skovde, SE-541 28 Skovde, Sweden; Medical Molecular Genetics Department, Human Genetics and Genome Research Institute, National Research Centre, 12622 Cairo, Egypt; College of Medicine, University of Baghdad, 10071 Baghdad, Iraq; Children Welfare Teaching Hospital, 10071 Baghdad, Iraq; Genetics and Precision Medicine department, King Abdullah Specialized Children’s Hospital, King Abdulaziz Medical City, Ministry of National Guard Health Affairs, 22384 Riyadh, Saudi Arabia; King Abdullah International Medical Research Center, King Saud Bin Abdulaziz University for Health Sciences, Ministry of National Guard Health Affairs, 22384 Riyadh, Saudi Arabia; Department of Zoology, College of Science, King Saud University, 11421 Riyadh, Saudi Arabia; Department of Translational Genomics, Center for Genomic Medicine, King Faisal Specialist Hospital and Research Center, 12713 Riyadh, Saudi Arabia; Centogene GmbH, 18055 Rostock, Germany; Department of Cellular and Molecular Medicine, WJC PANUM, University of Copenhagen, DK-1165 Copenhagen, Denmark; Centre for Biotechnology and Microbiology, University of Swat, Swat 19120, Pakistan; Pediatric Neurology Department Pediatric Ward Faculty of Medicine, Mashhad University of Medical Sciences, Mashhad, Iran; Institute of Human Genetics, Medical University Innsbruck, 6020 Innsbruck, Austria; Department of Pediatrics, North Khorasan University of Medical Sciences, Bojnurd, Iran; Research Programs Unit, Stem Cells and Metabolism, Faculty of Medicine, University of Helsinki, 00014 Helsinki, Finland; Department of Child Neurology, Children’s Hospital, Paediatric Research Center, University of Helsinki and Helsinki University Hospital, 00014 Helsinki, Finland; Human Molecular Genetics Laboratory, Health Biotechnology Division, National Institute for Biotechnology and Genetic Engineering (NIBGE) College, PIEAS, 44000 Faisalabad, Pakistan; Centre for Regenerative Medicine and Stem Cell Research, Juma Building, Aga Khan University, Karachi 74800, Pakistan; Human Molecular Genetics Laboratory, Health Biotechnology Division, National Institute for Biotechnology and Genetic Engineering (NIBGE) College, PIEAS, 44000 Faisalabad, Pakistan; Genetics and Precision Medicine department, King Abdullah Specialized Children’s Hospital, King Abdulaziz Medical City, Ministry of National Guard Health Affairs, 22384 Riyadh, Saudi Arabia; Division of Genetic & Genomic Medicine, Nationwide Children’s Hospital, Columbus, OH 43205, USA; Department of Pediatrics, The Ohio State University College of Medicine, Columbus, OH 43210, USA; Genetics Institute and Genomic Center, Tel Aviv Sourasky Medical Center, and Faculty of Medicine, Tel Aviv University, Tel-Aviv, Israel; Nephro-Genetic Clinic, Nephrology Department and Genetics Institute, Tel Aviv Medical Center, Tel Aviv 64239, Israel; Molecular Neurosciences, Developmental Neurosciences, Zayed Centre for Research into Rare Disease in Children, UCL Great Ormond Street Institute of Child Health, London WC1N 1EH, UK; Department of Neurology, Great Ormond Street Hospital, London WC1N 1EH, UK; Department of Molecular and Human Genetics, Baylor College of Medicine, Houston, TX 77030, USA; Department of Pediatrics, Faculty of Medicine, Kuwait University, Safat 13110, Kuwait; Narges Medical Genetics and Prenatal Diagnosis Laboratory, Kianpars, Ahvaz, Iran; Department of Medical Genetics, Faculty of Medicine, Ahvaz Jundishapur University of Medical Sciences, Ahvaz, Iran; Department of Molecular Medicine, Biotechnology Research Center, Pasteur Institute of Iran, Tehran, Iran; Nephro-Genetic Clinic, Nephrology Department and Genetics Institute, Tel Aviv Medical Center, Tel Aviv 64239, Israel; Molecular Neurosciences, Developmental Neurosciences, Zayed Centre for Research into Rare Disease in Children, UCL Great Ormond Street Institute of Child Health, London WC1N 1EH, UK; Department of Neurology, Great Ormond Street Hospital, London WC1N 1EH, UK; Department of Neurology, Great Ormond Street Hospital, London WC1N 1EH, UK; Developmental-Behavioural Paediatrics Department, University of Child Health Sciences & The Children’s Hospital, 54000 Lahore, Pakistan; Developmental-Behavioural Paediatrics Department, University of Child Health Sciences & The Children’s Hospital, 54000 Lahore, Pakistan; Center for Social Pediatrics and Epilepsy Outpatient Clinic Frankfurt Mitte, 60316 Frankfurt am Main, Germany; GeneDx, Gaithersburg, MD 20877, USA; Department of Neuromuscular Diseases, University College London, Queen Square, Institute of Neurology, London WC1N 3BG, UK; Department of Neuromuscular Diseases, University College London, Queen Square, Institute of Neurology, London WC1N 3BG, UK; Department of Neurology, Feinberg School of Medicine, Northwestern University, Chicago, IL 60611, USA; Institute of Medical Genetics, University of Zurich, 8952 Schlieren, Switzerland; Institute of Medical Genetics, University of Zurich, 8952 Schlieren, Switzerland; Institute of Medical Genetics, University of Zurich, 8952 Schlieren, Switzerland; Department of Medical Genetics, Istanbul Faculty of Medicine, Istanbul University, 34093 Istanbul, Turkey; Department of Medical Genetics, Istanbul Faculty of Medicine, Istanbul University, 34093 Istanbul, Turkey; Department of Neuromuscular Diseases, University College London, Queen Square, Institute of Neurology, London WC1N 3BG, UK; Department of Biotechnological and Applied Clinical Sciences (DISCAB), University of L’Aquila, 67100 L’Aquila, Italy; Human Molecular Genetics Laboratory, Health Biotechnology Division, National Institute for Biotechnology and Genetic Engineering (NIBGE) College, PIEAS, 44000 Faisalabad, Pakistan; Clalit Research Institute, Clalit Health Services, 6578898 Ramat Gan, Israel; Faculty of Medicine, Tel Aviv University, Tel Aviv, Israel; School of Health Science, Division Biomedicine and Translational Medicine, University of Skovde, SE-541 28 Skovde, Sweden; Department of Pediatrics, Prince Sultan Military Medical City, 12233 Riyadh, Saudi Arabia; American University of Beirut, 1107 2020 Beirut, Lebanon; Alfaisal University, 11533 Riyadh, Saudi Arabia; Division of Medical Genetics, Massachusetts General Hospital, Boston, MA 02114, USA; Harvard Medical School, Boston, MA 02115, USA; Harvard Medical School, Boston, MA 02115, USA; Department of Pediatric Neurology, Massachusetts General Hospital, Boston, MA 02114, USA; Department of Biology, Faculty of Science, Shahid Chamran University of Ahvaz, Ahvaz, Iran; Department of Biology, Faculty of Science, Shahid Chamran University of Ahvaz, Ahvaz, Iran; Narges Medical Genetics and Prenatal Diagnosis Laboratory, Kianpars, Ahvaz, Iran; Narges Medical Genetics and Prenatal Diagnosis Laboratory, Kianpars, Ahvaz, Iran; Ati Mehr Kasra Genetics Institute, Kianpars, Ahvaz, Iran; Narges Medical Genetics and Prenatal Diagnosis Laboratory, Kianpars, Ahvaz, Iran; Department of Medical Genetics, Faculty of Medicine, Ahvaz Jundishapur University of Medical Sciences, Ahvaz, Iran; Narges Medical Genetics and Prenatal Diagnosis Laboratory, Kianpars, Ahvaz, Iran; Diabetes Research center, Health Research Institute, Ahvaz Jundishapur University of Medical Sciences, Ahvaz, Iran; Department of Pediatrics Endocrinology, Mashhad University of Medical Sciences, Mashhad, Iran; Specialized Immunology Laboratory of Dr Shahrooei, Sina Medical Complex, Ahvaz, Iran; Department of Microbiology and Immunology, Clinical and Diagnostic Immunology, KU Leuven, 3000 Leuven, Belgium; Centogene GmbH, 18055 Rostock, Germany; Cerebellar Malformations and Congenital diseases Reference Center and Neurogenetics Lab, Department of Genetics, Armand Trousseau Hospital, AP-HP Sorbonne Université, 75006 Paris, France; Developmental Brain Disorders Laboratory, Imagine Institute, INSERM UMR 1163, 75015 Paris, France; Pediatric Neurology Department, Movement Disorders Center, Armand Trousseau Hospital, AP-HP Sorbonne Université, 75006 Paris, France; Institute of Human Genetics, Medical University Innsbruck, 6020 Innsbruck, Austria; Institute of Human Genetics, Medical University Innsbruck, 6020 Innsbruck, Austria; Institute of Human Genetics, Klinikum rechts der Isar, Technische Universität Munich, 81675 Munich, Germany; Hull York Medical School, Hull HU6 7RX, UK; Department of Neurology, Feinberg School of Medicine, Northwestern University, Chicago, IL 60611, USA; Department of Neurology, Faculty of Medicine, Beni-Suef University, 62521 Beni Suef, Egypt; Center for Human Genetics, University Hospitals Leuven, 3000 Leuven, Belgium; Laboratory for the Genetics of Cognition, Department of Human Genetics, KU Leuven–University of Leuven, 3000 Leuven, Belgium; Department of Pediatric Neurology, University Children's Hospital Zürich, University of Zürich, 8032 Zürich, Switzerland; Wellcome Centre for Human Genetics, University of Oxford and Oxford NIHR Biomedical Research Centre, Oxford, OX3 7BN UK; Departments of Pediatrics, College of Medicine and Health Sciences, United Arab Emirates University, Al Ain, UAE; Centogene GmbH, 18055 Rostock, Germany; Department of Neurosciences, University of California, San Diego, La Jolla, CA 92093, USA; Rady Children’s Institute for Genomic Medicine, San Diego, CA 92025, USA; Department of Translational Genomics, Center for Genomic Medicine, King Faisal Specialist Hospital and Research Center, Riyadh 11211, Saudi Arabia; Department of Molecular and Human Genetics, Baylor College of Medicine, Houston, TX 77030, USA; Texas Children’s Hospital, Houston, TX 77030, USA; Human Genome Sequencing Center, Baylor College of Medicine, Houston, TX 77030, USA; Department of Biology, Faculty of Science, Shahid Chamran University of Ahvaz, Ahvaz, Iran; Ati Mehr Kasra Genetics Institute, Kianpars, Ahvaz, Iran; Department of Pediatric Neurology, Golestan Medical, Educational, and Research Center, Ahvaz Jundishapur University of Medical Sciences, Ahvaz, Iran; Boston Children’s Hospital and Harvard Medical School Boston, MA 02115, USA; Human Molecular Genetics Laboratory, Health Biotechnology Division, National Institute for Biotechnology and Genetic Engineering (NIBGE) College, PIEAS, 44000 Faisalabad, Pakistan; Department of Biological and Biomedical Sciences, Aga Khan University, 74800 Karachi, Pakistan; Department of Neuromuscular Diseases, University College London, Queen Square, Institute of Neurology, London WC1N 3BG, UK; Neuroradiology Unit, IRCCS Istituto Giannina Gaslini, 16147 Genoa, Italy

**Keywords:** mediator complex, gene transcription, neurodevelopmental disorders, dystonia, cerebello-lental degeneration, cerebellar atrophy

## Abstract

MED27 is a subunit of the Mediator multiprotein complex, which is involved in transcriptional regulation. Biallelic *MED27* variants have recently been suggested to be responsible for an autosomal recessive neurodevelopmental disorder with spasticity, cataracts and cerebellar hypoplasia. We further delineate the clinical phenotype of *MED27*-related disease by characterizing the clinical and radiological features of 57 affected individuals from 30 unrelated families with biallelic *MED27* variants.

Using exome sequencing and extensive international genetic data sharing, 39 unpublished affected individuals from 18 independent families with biallelic missense variants in *MED27* have been identified (29 females, mean age at last follow-up 17 ± 12.4 years, range 0.1–45). Follow-up and hitherto unreported clinical features were obtained from the published 12 families. Brain MRI scans from 34 cases were reviewed.

*MED27*-related disease manifests as a broad phenotypic continuum ranging from developmental and epileptic-dyskinetic encephalopathy to variable neurodevelopmental disorder with movement abnormalities. It is characterized by mild to profound global developmental delay/intellectual disability (100%), bilateral cataracts (89%), infantile hypotonia (74%), microcephaly (62%), gait ataxia (63%), dystonia (61%), variably combined with epilepsy (50%), limb spasticity (51%), facial dysmorphism (38%) and death before reaching adulthood (16%). Brain MRI revealed cerebellar atrophy (100%), white matter volume loss (76.4%), pontine hypoplasia (47.2%) and basal ganglia atrophy with signal alterations (44.4%). Previously unreported 39 affected individuals had seven homozygous pathogenic missense *MED27* variants, five of which were recurrent. An emerging genotype-phenotype correlation was observed.

This study provides a comprehensive clinical-radiological description of *MED27*-related disease, establishes genotype-phenotype and clinical-radiological correlations and suggests a differential diagnosis with syndromes of cerebello-lental neurodegeneration and other subtypes of ‘neuro-MEDopathies’.

## Teaser

Biallelic variants in MED27 are associated with an ultra-rare neurodevelopmental disorder with spasticity, cataracts and cerebellar hypoplasia. Maroofian *et al*. further delineate the clinical phenotype of MED27-related disease by characterizing the clinical and radiological features of 57 affected individuals from 30 unrelated families.

## Introduction

Mediator (MED) is a large, evolutionarily conserved multiprotein complex that plays a critical role in facilitating transcriptional regulation by acting as a physical and functional bridge between DNA-binding transcription factors and the transcriptional machinery. Mediator comprises ∼25 subunits in yeast and ∼30 distinct subunits in humans that are organized into three main modules: head, middle and tail, forming the core MED, and a separable four-subunit kinase module (CKM).^1,2^

The biological significance of Mediator in human development is vigorously demonstrated by an emerging list of human pathologies linked to genetic variation or aberrant expression of genes encoding the individual subunits. Pathogenic variant alleles in several of the genes for MED complex subunits are associated with distinct monogenic neurodevelopmental or neurodegenerative disorders (NDDs) with overlapping clinical features, which could collectively be grouped under the umbrella term ‘MEDopathies’ or ‘Neuro-MEDopathies’. Pathogenic variants in *MED17* (MIM:603810), *MED20* (MIM:612915), *MED23* (MIM:605042), *MED25* (MIM:610197) and *MED11* (MIM:612383) lead to autosomal recessive NDDs.^3-7^*De-novo*/heterozygous pathogenic variants in *MED12L* (MIM:611318), *MED13* (MIM: 603808), *MED13L* (MIM:608771), *CDK8* (MIM:603184) and *CDK19* (MIM:614720) cause autosomal dominant NDDs.^8-12^ Hemizygous pathogenic variants in *MED12* (MIM:300188) are associated with multiple X-linked recessive intellectual disability (ID) syndromes, including Opitz–Kaveggia syndrome (MIM:305450), also known as FG syndrome, Lujan–Fryns syndrome (MIM:309520) and X-linked Ohdo syndrome (MIM:300895) as well as non-syndromic ID. Heterozygous *MED12* pathogenic variants cause an X-linked dominant multiple congenital anomaly disorder known as Hardikar syndrome which is only reported in females.^13^

The MED27 protein is a subunit of the mediator complex, which connects transcription factors to the RNA polymerase II complex; thus, it is an essential part of the eukaryotic transcription machinery. The MED27 protein, along with MED28, MED29 and MED30, forms the upper tail segment.^14^ It has recently been shown that biallelic *MED27* variants cause an NDD with spasticity, cataracts and cerebellar hypoplasia (MIM:219286).^15,16^ Here, we further characterize molecular and clinical-radiological features of a large cohort consisting of new and previously reported cases with biallelic likely pathogenic and pathogenic *MED27* variants.

## Subject and methods

### Patient ascertainment and clinical and genetic investigations

To further delineate *MED27*-related NDD, we applied a genotype-first approach by data mining large aggregates of DNA sequences from different diagnostic and research genetic laboratories, including Queen Square Genomics, Centogene, GeneDx, Baylor Genetics, Invitae, 100,000 Genomes Project, ClinVar, Decipher, DDD study, Geno2MP and many other local databases worldwide, as well as using GeneMatcher^17^ and VarSome. A detailed clinical proforma was completed by recruiting clinicians for unpublished and published affected individuals. Previously unreported features, some follow-up clinical data and brain MRI, including new MRI scans were obtained from all reported families. The institutional review boards of University College London as well as the respective host institution approved this study. All study participants and/or their parents or guardians signed informed consent allowing for participation and publishing of photographs and videos and other relevant identifying information.

Exome sequencing (ES) was performed on genomic DNA extracted from blood in different diagnostic or research laboratories around the world (Supplementary Table 1). The candidate variants were confirmed after filtering (i.e. minor allele frequency 0–0.005) and interpretation according to the American College of Medical Genetics and Genomics and the Association for Molecular Pathology (ACMG-AMP) guidelines,^18^ and segregation analysis was performed by Sanger sequencing.

### Neuroradiological review

Brain MRI studies were reviewed in consensus by two experienced paediatric neuroradiologists (M.S and D.T). Qualitative and semi-quantitative assessments of the cerebellar atrophy and supratentorial white matter volume reduction were performed as previously described.^19^ Additional neuroimaging information regarding cortical, basal ganglia, white matter and midbrain-hindbrain anomalies were noted. Continuous variables were summarized as mean, and categorical variables were summarized as frequencies and percentages. Age differences at the last brain MRI for clinical and neuroimaging features were tested using the Mann–Whitney U-test. The associations between clinical and neuroradiological findings were evaluated by the chi-squared and Fisher’s exact test. Statistical significance was set to *P* = 0.05. Statistical analyses were performed using SPSS Statistics software, v26 (IBM, Armonk, NY, USA) (see Supplementary material for details).

### Single state difference in Gibbs free energy calculations

The cryo-EM structure PDB:6W1S^14^ of the mouse mediator complex was used for analyses in PyRosetta^20^ (code available at https://github.com/matteoferla/MED27_analysis). The structure was energy minimized using three cycles of LocalRelax,^21^ followed by one cycle of FastRelax for each chain. To score each variant, the 10 Å neighbourhood of the residue affected was energy minimized (FastRelax) for five cycles before and after the introduction of the substitution using the ref2015 score function.^22^ The local flexibility of the structure was probed using the BackrubMover.^23^ Structures have been shared via Michelaɴɢʟo.^24^ Methods for multiple sequence alignment are provided in the Supplementary material.

## Results

### Genetic findings

**Figure 1:**
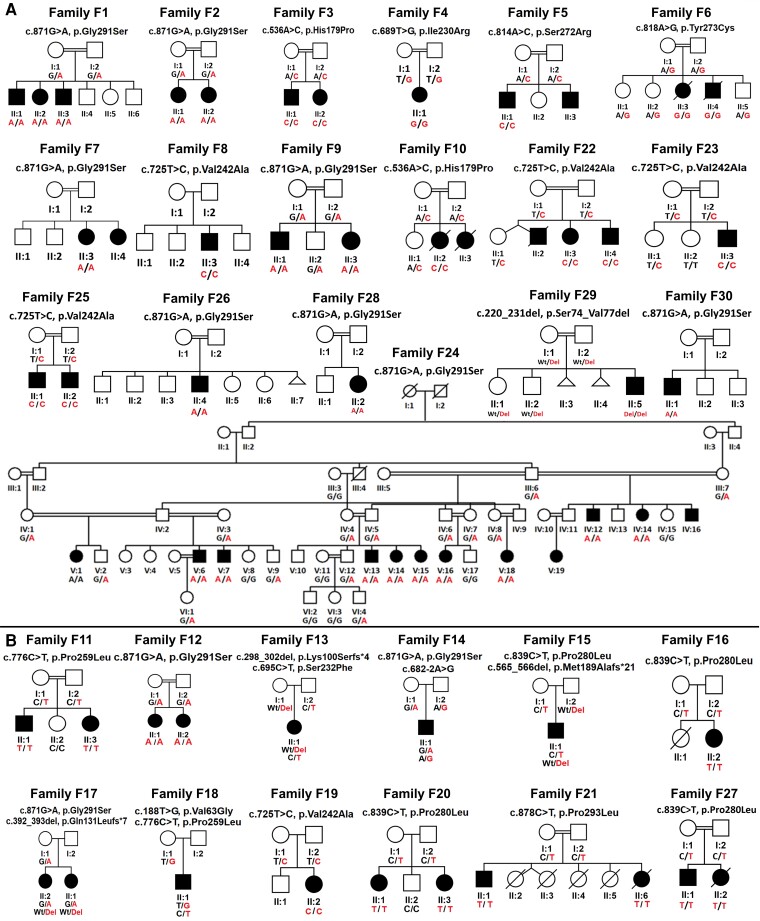
**Pedigrees of affected families showing segregation of the biallelic *MED27* variants**. (**A**) New families; (**B**) reported families. Filled symbol = affected. Genotype, where indicated, represents the results of the evaluation of the variants by Sanger sequencing.

**Figure 2:**
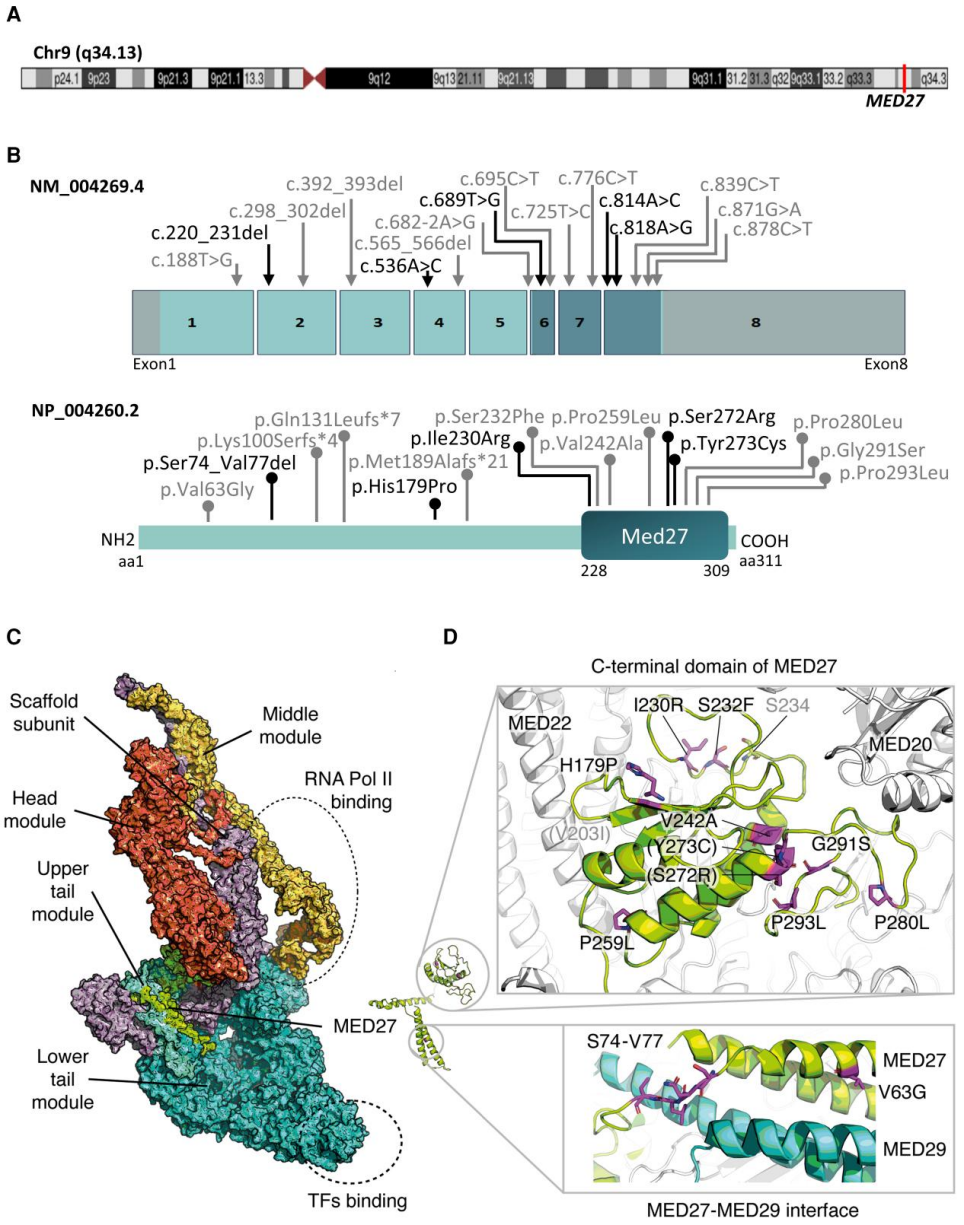
***In silico* modelling of *MED27* gene, protein and position of the variants**. (**A**) Chromosome 9 showing position of *MED27* on 9q34.13. (**B**) Schematic diagram of *MED27* gene and protein. The location of previously reported variants is indicated by a grey line and new variants by a black line. Introns are not to scale. Exon numbers are according to the canonical transcript (NM_021723.5). Amino acid changes are according to the reference sequence NP_004260.2. (**C**) Mediator complex (PDB:6W1S) coloured by module, with binding regions marked by dotted ellipses. The MED27 protein is coloured in green and is part of the lower tail module. (**D**) MED27 with residues that present a pathogenic variant are highlighted. Residues that are hidden are in parentheses. Those in grey are gnomAD variants of interest (for interactive view: https://michelanglo.sgc.ox.ac.UK/r/med27).

We identified 39 unpublished affected individuals across 18 families harbouring eight homozygous *MED27* variants, five of which have not previously been reported. The variants were as follows: Families 1, 2, 7, 9, 24, 26, 28 and 30, c.871G>A, p.Gly291Ser; Families 3 and 10, c.536A>C, p.His179Pro; Family 4, c.689T>G, p.Ile230Arg; Family 5, c.814A>C, p.Ser272Arg; Family 6, c.818A>G, p.Tyr273Cys; Families 8, 22, 23 and 25, c.725T>C, p.Val242Ala; and Family 29, c.220-231del, p.Ser74_Val77del (Figs 1, 2A and B and Supplementary Table 1). All variant alleles identified in the families were either absent or observed only as heterozygous at extremely low frequencies in over 1.5 million alleles across multiple publicly available and private genetic variant databases (range 0–0.003). Five variants (5/16, 31%) were observed in more than one family. Notably, p.Gly291Ser and p.Val242Ala variants were found in 10 independent Arab families and five independent Turkish/Aramaic families, respectively. Interestingly, haplotype analysis of the p.Gly291Ser revealed at least two distinct haplotypes surrounding the variant, suggesting two independent mutational events within Arabian Middle Eastern populations. On the other hand, the p.His179Pro variant identified in two independent Pakistani families showed similar haplotypes, indicating a possible founder effect for the variant (Supplementary Fig. 1A). In addition, all variants occurred in conserved amino acid residues in MED27 and segregated with disease within the families (Fig. 1 andSupplementary Fig. 1B). Computational variant prediction tools such as MutationTaster, SIFT and PolyPhen predict the functional impact of all but two variants as mainly damaging and deleterious. According to the American College of Medical Genetics and Genomics and the Association for Molecular Pathology (ACMG-AMP) system for variant classification,^18^ all the variants are classified as either likely pathogenic or pathogenic. The characteristics of the previously reported and new variants are summarized in Supplementary Table 1.

### *In silico* modelling

MED27 is composed of two regions, an N-terminal region that forms a heterodimeric helical bundle with MED29, and a C-terminal globular domain that interacts with various head segment proteins (Fig. 2C and D).^14^ Except for one variant allele, the translated products of the variants affect residues within the globular domain. The only variant that affects the MED27-MED29 heterodimeric region is p.Val63Gly, which is a structurally destabilizing substitution, affecting a residue close to the interface with MED29 (Fig. 2D). The majority of the variants in the globular domain are predicted to be destabilizing in the conformation analysed (ΔΔG > +2 kcal/mol). As a reference for variants that are potentially neutral, gnomAD, a database of variants from the healthy population, was inspected. There are only three stop-gain/frameshift and 42 missense variants reported in the canonical transcript (Q6P2C8, ENST00000292035, NM_004269, 311 amino acids long), which is the sole transcript in the tissues reported in the GTex database.^25^ Of those, only three variants are predicted to be destabilizing. The remaining variants are either structurally neutral or occur in amino acids within the regions of missing density (Supplementary material).

### Clinical delineation

**Figure 3:**
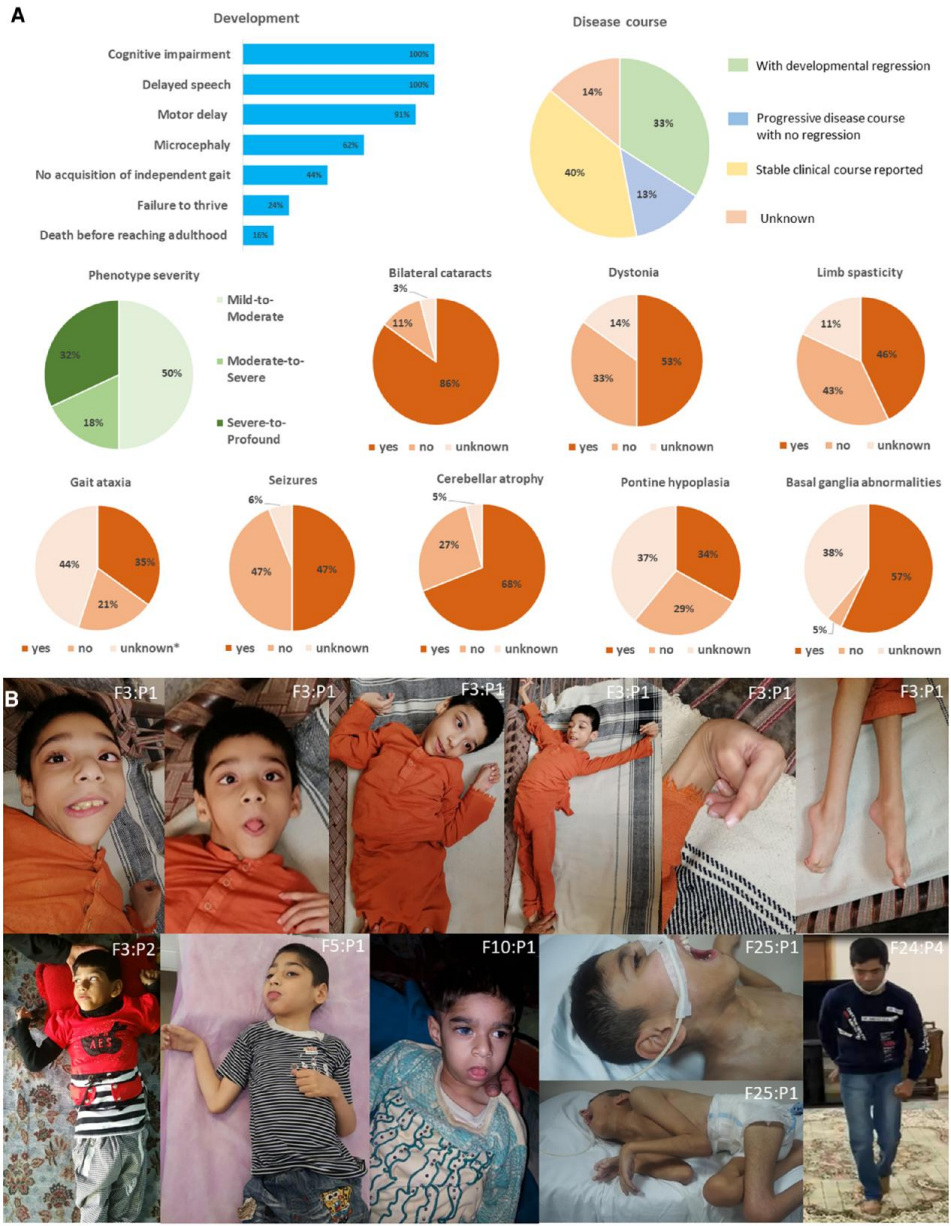
**Clinical features of *MED27*-related disease**. (**A**) The most frequent clinical features. (**B**) Representative photographs. *Top*: Patient F3:P1 with generalized dystonia. *Bottom* from *left* to *right*: Patient F3:P2 with flexed and raised arms in elbows with shoulder extension, a suggestion of tongue protrusion; F5:P1 with flexed upper limbs in elbows and wrists and tongue protrusion; F10:P1 with tongue protrusion and cataract, visible in his right eye; F25:P1 with flexion contractures, generalized muscle wasting and a suggestion of jaw-opening dystonia; Patient F24:P4 with generalized dystonia involving the limbs and trunk.

Clinical data were assembled from 18 unpublished independent families with 39 affected individuals along with follow-up clinical details from 18 reported individuals across 12 families, and the summary is presented in Table 1, Fig. 3 and Supplementary Table 2. Overall, 57 patients from 30 independent families were included. Video recordings were available from Families 3, 5, 11, 24, 25, 26, 28, 29 and 30 (Supplementary Videos 1–9, available at FigShare doi: 10.6084/m9.figshare.23723940).

**Table 1:** Main clinical features of the affected individuals with biallelic variants in MED27

Category/clinical feature	*n* (percentage)
Sex	28 males, 29 females
Consanguinity	21/30 (70%)
Mean age at the most recent follow-up, years	17 ± 12.4 (range 0.1–45)
Abnormal prenatal and perinatal history	6/55 (11%), NA 2
Mortality	9/57 (16%)
Failure-to-thrive	11/47 (24%), NA 10
Microcephaly	24/39 (62%), NA 18
Cognitive impairment/ID	54/55 (98%), NA 2
Severity of ID	38% mild; 30% moderate-to-severe; 21% severe-to-profound; 10% NA
Hypotonia in infancy	32/43 (74%), NA 14
Motor delay	48/53 (91%), NA 4
No acquisition of independent gait	23/52 (44%), NA 5
Speech delay/non-verbal	53/53 (100%), NA 4
Able to perform ADLs	17/49 (35%), NA 8
Regression	19/52 (37%), NA 5
Progressive course	20/47 (43%), NA 10
Dysmorphic features	20/52 (38%), NA 5
Bilateral cataracts	49/55 (89%), NA 2
Seizures	27/54 (50%), NA 3
Mean age of seizure onset, years	6.2 ± 2.3 (range 0.1–10)
Dysarthria	19/29 (66%), NA 28
Hypotonia at follow-up	28/51 (55%), NA 9
Spasticity	26/51 (51%), NA 6
Cerebellar ataxia	20/62 (63%), NA2 5
Dystonia/dyskinesia	30/49 (61%), NA 8
Joint contractures	20/52 (38%), NA 5
Cerebellar atrophy	36/36 (100%); NA 21; mild 22/36 (61%); moderate-to-severe 14/36 (39%)
Pontine hypoplasia	17/36 (47%)
Basal ganglia abnormalities	32/36 (89%)

ADL = activities of daily living; ID = intellectual disability; NA = not available or not applicable/unable to assess.

The cohort comprised 29 females and 28 males with a mean age of 17 ± 12.4 years (age range 2 months–45 years) at the most recent follow-up. Nine affected individuals died at a mean age of 5.9 ± 4.8 years (age range 2 months–13 years).

Prenatal and perinatal histories were mostly unremarkable (50/54, 93%) with full-term birth in 30/36 cases (83%), while only six affected individuals had abnormal antenatal findings (Table 1 and Supplementary Table 2 for details). Failure to thrive was reported in 11/47 (24%) patients.

Infantile hypotonia (32/43, 74%) and global developmental delay (GDD), including motor delay (48/53, 91%), delayed cognitive development ranging from mild to profound (50/50, 100%) and delayed speech (53/53, 100%), were common disease manifestations. Age at unsupported sitting was available in 36 cases, 17 of whom had delayed sitting, while 16 never attained independent sitting. Age at walking was obtainable in 41 cases, 23 of whom have failed to attain independent walking, eight walked at a normal age, and 10 acquired unassisted walking between the ages of 19 and 38 months. Twenty of 53 cases with delayed speech were completely non-verbal. Only 35% (17/49) of the affected individuals were able to perform basic activities of daily living (ADLs). The loss of acquired milestones was reported in 37% of cases (19/52) and a progressive disease course was confirmed in 20/47 (43%) affected individuals.

At the latest available follow-up, weight and height were below the 10th percentile in 24/37 (65%) and 17/35 (49%) cases, respectively. Microcephaly was present in 24/39 (62%) cases. The common neurological features included gait ataxia (20/32, 63%), central hypotonia (28/51, 55%) and dystonia (30/49, 61%) combined with excessive drooling (19/36, 53%), epilepsy (27/54, 50%), hyperreflexia (23/40, 58%) and limb spasticity (26/51, 51%). In 20/50 (40%) cases, it was not possible to assess cerebellar ataxia due to either infantile mortality or lack of independent ambulation, and severe cognitive impairment variably combined with generalized dystonia. Dysarthria was confirmed in 19 of 29 individuals with delayed speech acquisition. Dystonia was generalized in 17/38 (45%) cases, occasionally leading to opisthotonic posture of the trunk, associated with fixed dystonia in the limbs, and involving the tongue (12/38, 32%) and face/jaw (10/34, 30%). Dystonia was responsive to L-dopa in two cases from Family 12. Spasticity had variable severity and equally affected upper and lower limbs in 14 cases, while seven affected individuals were confirmed to have predominantly lower limb spasticity. Less frequent, but present in around one-third of cases, were limb contractures (20/52, 38%), dyskinetic movements (11/41, 27%) and strabismus (8/43, 19%).

Seizures manifested from the first hours of life up to the age of 10 years, but most were of infantile or early childhood onset. Seizure types varied from infantile spasms, absences and myoclonic seizures to focal motor, tonic and generalized tonic-clonic seizures (Supplementary Table 2). Some cases had seizures with different semiology. Seizure frequency and responses to antiepileptic medications were also variable, ranging from multiple daily episodes to once every 3 months, along with a good response to either phenobarbital, levetiracetam or valproate or a combination thereof. Some cases had medically intractable seizures despite polytherapy. Twenty patients (36%) presented with a combination of epilepsy/epileptic encephalopathy and dystonia/dyskinesia.

Most of the subjects developed bilateral cataracts (49/55, 89%), which were confirmed to be congenital in 11/19 (58%) cases. Most of the affected individuals (40/47, 85%) underwent cataract surgery.

Variable non-specific dysmorphic features, frequently including low set ears, triangular face, micrognathia, low anterior hairline and short philtrum, were present in 20/52 (38%) patients (Supplementary Table 2).

Among the miscellaneous features were urinary/bowel incontinence (16/42 38%), respiratory problems (13/47, 28%), hyperactivity and aggression (6/38, 16%), feeding difficulties (16/47, 34%) and scoliosis (6/57, 11%). Single individuals developed bilateral hearing impairment, upper limb coarse tremor and autism.

### Genotype-phenotype correlation

Three phenotype severity levels were distinguished in the cohort based on the degree of GDD/ID and the presence of early death, microcephaly, regression and epilepsy in combination with the presence and severity of cerebellar ataxia and dystonia, and ADLs. The phenotype severity was mild-to-moderate in 29/57 (51%), moderate-to-severe in 10/57 (17%) and severe-to-profound in 18/57 (32%) families leading to early death (before reaching adulthood) in 9/57 (16%) cases. Five *MED27* variants in a homozygous or compound heterozygous state recurred in 24 families (Table 1, Supplementary Table 1 and Fig. 2). Homozygous carriers of the most recurrent *MED27* variant, c.871G>A, p.Gly291Ser (eight families from the Middle East), and cases with the c.776C>T, p.Pro259Leu variant predominantly had a mild-to-moderate phenotype. Whilst two unrelated cases harbouring the *MED27* c.536A>C, p.His179Pro variant presented a severe-to-profound phenotype, eight affected individuals from five independent Turkish families harbouring the recurrent homozygous *MED27* c.725T>C, p.Val242Ala variant and individuals carrying the *MED27* c.839C>T, p.Pro280Leu variant demonstrated moderate-to-severe and severe-to-profound phenotypes. There were four patients who carried biallelic *MED27* variants, one of which was a truncating variant. All these patients fell in the category of moderate-to-severe phenotype.

### Neuroimaging findings and clinical-neuroradiological correlation

**Figure 4:**
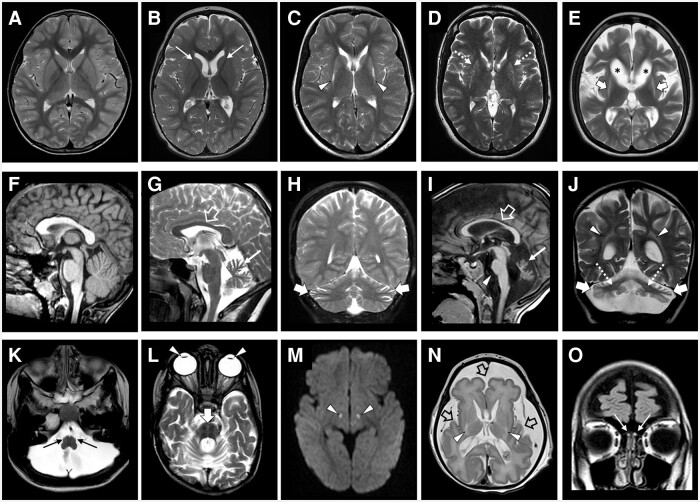
**Neuroradiological features of *MED27-*related disease**. Brain MRI images showing the spectrum of basal ganglia anomalies (**A**–**E**) and cerebellar atrophy (**F**–**J**), with a normal subject for comparison (**A** and **F**), and of other less frequent neuroimaging findings in the study cohort (**K**–**O**). (**A**–**E**) Axial T_2_-weighted images reveal mild volume reduction of the caudate nuclei in Patient F19:P1 (thin arrows, **B**); moderate atrophy of both the caudate and putamen nuclei associated with bilateral hyperintensity of the posterior putamen in Patient F24:P7 (arrowheads, **C**); bilateral moderate atrophy with subtle hyperintensity of the putamen and caudate nuclei in Patient F12:P2 (dashed arrows, **D**); and severe atrophy with marked hyperintensity of the same nuclei in F3.P1 (thick arrows, **E**). In addition, Patient F3:P1 shows severe white matter volume loss, with enlargement of the lateral ventricle (asterisks, **E**) and enlargement of the cerebral subarachnoid spaces. (**G** and **H**) Sagittal and coronal T_2_-weighted images demonstrate mild cerebellar atrophy in Patient F24:P2, with prevalent superior vermian involvement (thin arrow, **G**) and less severe subarachnoid spaces enlargement of the lateral portions of the cerebellar hemispheres (thick arrows, **H**). The corpus callosum is slightly thin (open arrow, **G**). (**I** and **J**) Sagittal T_1_-weighted and coronal T_2_-weighted images show severe cerebellar atrophy in Patient F20:P2, with prevalent superior vermian involvement (thin arrow, **I**), marked T_2_-hyperintensity of the cerebellar cortex (thick arrows, **J**), pontine hypoplasia (arrowhead, **J**) and faint T_2_-hyperintensity of the dentate nuclei (dashed arrows, **J**). There is moderate periventricular white matter volume loss and T_2_-hyperintensity (arrowheads, **J**) with a thin corpus callosum (open arrow, **I**). (**K**) Axial T_2_-weighted image demonstrates volume reduction and faint hyperintensity of the inferior olivary nuclei in Patient F3:P1 (thin arrows). (**L**) Axial T_2_-weighted image shows hyperintensity forming a cross in the pons (hot cross bun sign) due to selective degeneration of the transverse pontocerebellar tracts and median pontine raphe nuclei in Patient F20:P2 (thick arrow). Note the presence of bilateral intraocular lens implants (pseudophakia) as a result of the surgical treatment for cataract (arrowheads). (**M**) Axial diffusion-weighted image reveals bilateral small focal hyperintensities consistent with restricted diffusion at the level of the central midbrain and subthalamic nuclei in Patient F9:P2 (arrowheads). (**N**) Axial T_2_-weighted image demonstrates microcephaly with marked simplification of the cortical gyral pattern in Patient F20:P (open arrows). The caudate and putamen nuclei are very small with bilateral focal hyperintensity of the posterior portions of the putamina (arrowheads). (**O**) Coronal fluid-attenuated inversion recovery image depicts bilateral atrophy and hyperintensity of the olfactory bulbs in Patient F9:P1 (thin arrows).

Neuroimaging studies were available for review in 36/57 (63.1%) subjects. Follow-up MRI studies were performed in 12/36 (33.3%) subjects, with a total number of 51 MRI scans available for review. The mean age at MRI was 7.7 years (range 1 day—37 years). The mean follow-up duration was 4.4 years (range 1–15 years). Neuroimaging features and clinical-neuroimaging associations are detailed in Fig. 4, Table 1, Supplementary Tables 2–5 and Supplementary Figs 3–6. All subjects had cerebellar atrophy with prevalent vermian involvement and variable severity: very mild/mild in 22/36 (61.6%) and moderate-severe in 14/36 (38.8%); in 7/36 (19.4%) cases, there was additional cerebellar cortex T_2_ hyperintensity. Pontine hypoplasia was observed in 17/36 (47.2%) cases. Additional posterior cranial fossa features were cerebellar dentate nuclei T_2_ hyperintensities (8/36, 22.2%), hot cross bun sign (3/36, 8.3%) and olivary nuclei degeneration (3/36, 8.3%). Severe caudate nuclei and putamen volume reduction were noted in 15/36 (41.6%) and 17/36 (47.2%) of cases, respectively. Symmetric T_2_ signal alterations of the caudate nuclei and putamen were present in 11/36 (30.5%) and 16/36 (44.4%) cases. Overall, significant basal ganglia atrophy associated with T_2_ signal alterations was present in 16/36 subjects (44.4%). Mild-to-severe white matter volume reduction with ventricular enlargement was detected in 27/36 (75%) subjects, with associated T_2_ signal alterations and thin corpus callosum in 19/36 (52.7%) and 13/36 (36.1%) of cases, respectively. Mild-to-moderate enlargement of the cerebral CSF spaces was noted in 25/36 patients (69.4%). Mild simplification of the gyral pattern was detected in 13/36 (36.1%) subjects. Olfactory bulb-T_2_ signal alterations were visible in 5/36 (13.8%) subjects. Periventricular nodular heterotopias were noted in 2/36 (5.5%) cases, while Leigh-like signal alterations with restricted diffusion in the brainstem were present in two other subjects (5.5%) (Supplementary Fig. 5). Supplementary Table 4 reports in detail the evolution of neuroimaging features in the 12 subjects with follow-up MRI studies. In summary, all cases (100%) exhibited a progression of cerebellar atrophy, while 9/12 cases (75%) showed progressive white matter volume loss and/or basal ganglia atrophy. The progression apparent according to serial MRI studies is consistent with neurodegeneration.

A severe-to-profound clinical phenotype was associated with presence of moderate-severe cerebellar atrophy (*P* < 0.001), white matter volume reduction (*P* = 0.002) and thin corpus callosum (*P* = 0.011). Subjects who never achieved independent walking more frequently showed moderate-to-severe cerebellar atrophy (*P* = 0.001), cerebellar cortex T_2_ hyperintensities (*P* = 0.004), cerebellar dentate nuclei signal alterations (*P* = 0.028), white matter volume loss (*P* = 0.009), white matter signal alterations (*P* = 0.024), thin corpus callosum (*P* = 0.007) and enlarged CSF spaces (*P* = 0.045). Dystonia/dyskinesia imaging was associated with white matter volume loss (*P* = 0.016) and enlarged CSF spaces (*P* = 0.049). No significant associations were found between ataxia and neuroimaging features.

Finally, we found that subjects with basal ganglia atrophy and signal alterations had an older age at last MRI than subjects without basal ganglia abnormalities (median age 9 years versus 4 years, *P* = 0.033).

## Discussion

This case series delineates the phenotype of *MED27*-related disease by characterizing the clinical-radiological features of 57 affected individuals from 30 unrelated families with biallelic *MED27* variants. The current cohort exhibited an early-infantile/early childhood-onset concomitant neurodevelopmental and neurodegenerative disorder with a complex neurological phenotype. Mild to profound GDD associated with bilateral cataracts and progressive movement abnormalities, mostly including cerebellar ataxia, dystonia and spasticity, with underlying cerebellar atrophy, variable pontine involvement and basal ganglia abnormalities on neuroimaging are the core phenotypes of *MED27*-related disease. The spectrum of severity of the rare disease trait phenotype was broad ranging from profound and progressive cognitive and/or motor disability resulting in early mortality to borderline ID with impaired expressive language, mild-to-moderate motor impairment and survival into adulthood. A significant interfamilial phenotypic heterogeneity, as well as some intrafamilial variability, was observed in the cohort, consistent with the ‘Clan Genomics’ hypothesis.^26^ Whilst some cases were non-ambulatory and non-verbal with severe-to-profound GDD/ID and generalized dystonia, other cases were able to perform ADLs with no dystonia and only mild gait imbalance, speech impairment and borderline ID. A clear genotype-phenotype correlation in the present cohort was observed (Supplementary Fig. 2).

The near absence of destabilizing *MED27* variants in the healthy population as reported in gnomAD indicates that destabilizing variants are not tolerated. Conversely, the variants in the cohort are mostly destabilizing and in the C-terminal domain, suggesting the mechanism for pathogenicity is partial loss-of-function (LoF). The absence of homozygous or compound heterozygous frameshift, nonsense and splicing *MED27* variants in all the inspected variant databases or in this cohort suggests these may be embryonically lethal, which is consistent with the animal model study and the hypothesis that transcription is not possible without a complete MED27 subunit. Interestingly, this absence of biallelic LoF variants was also observed in other subunits of the Mediator complex, suggesting that total loss of the complex is not viable. Further discussion points are provided in the Supplementary material. Differential diagnoses of *MED27*-related disease with other MEDopathies are provided in Supplementary Table 6.

The present cohort showed a recurring pattern of both brain and ocular involvement. Genetic syndromes involving both brain and eye abnormalities are numerous, and when the combination of cerebellar ataxia, dystonia and spasticity with bilateral cataracts is involved, the list of differential diagnoses narrows substantially. These mainly include but are not limited to Marinesco-Sjögren syndrome (MIM: 248800), autosomal recessive spastic paraplegia 46 (MIM:614409), Warburg micro syndrome (1 and 4) (MIM:600118, MIM: 614225, MIM: 614222, MIM: 615663) and cerebro-oculo-facio-skeletal syndrome (MIM: 214150). Suggested differential diagnoses for *MED*27-related disease are given in Supplementary Table 7. Whilst *MED27-*related disease has many overlapping clinical features with other oculocerebral syndromes, the most distinguishing feature of *MED27* defects is the prominence of dystonia. Various disease mechanisms could lead to cerebello-lental degeneration (Supplementary Table 7).

This study delineates the neuroradiological features of *MED27*-related disease. In particular, all subjects presented with cerebellar atrophy, which was further characterized by dentate nuclei and cerebellar cortex signal alterations in 22.2% and 19.4% of cases, respectively. In almost half of the cases, the pons was hypoplastic, with additional olivary nuclei degeneration and selective degeneration of transverse pontocerebellar tracts and median pontine raphe nuclei in 8.3% of subjects. These findings, together with the evidence of clear cerebellar atrophy progression on imaging in 12 subjects, favour the hypothesis of a prevalent neurodegenerative process. Thus, the cerebellar features in *MED27*-related disease are better defined as ‘atrophy’ rather than ‘hypoplasia’, as previously suggested.^13^ Moreover, the presence of pontine hypoplasia in a significant proportion of subjects with *MED27*-related disease might indicate an early, likely prenatal onset of cerebellar degeneration, as described in several ponto-cerebellar hypoplasias.^27,28^

Interestingly, the vast majority of subjects with *MED27*-related disease presented mild to severe white matter volume loss with thinning of the corpus callosum. These non-specific features associated with variable cerebellar atrophy have been described in other MEDopathies, including those related to variants in *MED*17,^3^*MED*20,^4^*MED*13L,^29,30^*CDK1*9^12^ and *MED11.*^7^ Of note, the degree and prevalence of cerebral atrophy greatly varies in *MED*-related diseases, ranging from the early onset and very severe atrophy described in all patients with *MED11* variants to milder and slowly progressive atrophy affecting about 70% of patients with *MED27* variants. Conversely, the peculiar combination of cerebellar atrophy and basal ganglia degeneration has been reported only in subjects with *MED20*-related disease, thus representing a helpful imaging pattern in the differential diagnosis of these conditions. Besides *MED20*-related disease, cerebellar atrophy with basal ganglia atrophy is a typical neuroimaging finding in hypomyelination with atrophy of the basal ganglia and cerebellum (H-ABC), Wilson’s disease (MIM:277900) and Huntington’s disease (MIM:143100).

Of note, in a subset of patients, we found malformations of cortical development, including diffuse cortical gyration simplification and periventricular nodular heterotopias. Interestingly, brain malformations have been reported in patients with other MEDopathies. In particular, corpus callosum agenesis, polymicrogyria and focal cortical dysplasias are described in *MED12L*,^8^*MED13L*,^29,30^*MED25*^6,31^ and *CDK8-*related disorders.^11^ This evidence raises the hypothesis of possible disruption of neurodevelopmental mechanisms often associated with neurodegenerative processes in several MEDopathies. *MED27* may have a role in both embryonic neuronal development and maintenance, later prenatally and in life. This could be the reason for the coexistence of neurodevelopmental abnormalities and neurodegeneration in the present cohort. Indeed, accumulating evidence suggests that proteins implicated in neurodegeneration play major roles in brain development.^32,33^

Regarding clinical-radiological correlations, we noticed that a moderate-to-profound clinical phenotype was associated with moderate-severe cerebellar atrophy and thin corpus callosum, thus supporting the hypothesis of a progressive neurodegenerative disorder. In particular, subjects who could not walk, compared with subjects presenting with ataxia, more frequently presented a severe neuroimaging pattern characterized by moderate-severe cerebellar atrophy, cerebellar cortex signal abnormalities, cerebellar dentate nuclei signal alterations, white matter volume loss and signal alterations, thin corpus callosum and brain atrophy. Interestingly, we did not find significant associations between dystonia/dyskinesia and basal ganglia anomalies. The fact that subjects with basal ganglia atrophy and signal alterations had an older age at last MRI than subjects without basal ganglia abnormalities might in part explain these discrepancies. Indeed, many patients in this study were scanned earlier in the disease course, while the last clinical assessment was frequently performed several years later. Longer follow-up MRI studies in subjects with *MED27-*related disease are needed to understand the association of basal ganglia degeneration and dystonia/dyskinesia.

*MED27*-related disease is an ultra-rare, complex neurodevelopmental syndrome variably associated with epilepsy-movement phenotype, in which one-third of patients present with developmental and epileptic-dyskinetic encephalopathy. Because of recent advances in high-throughput sequencing technologies, an ever-increasing number of NDDs manifesting with both epilepsy and progressive atypical movements are now recognized. It is proposed that this emerging ’progressive epilepsy-dyskinesia syndrome’ spectrum is attributed to the genes encoding proteins with a diverse set of neuronal functions,^34^ which could underscore the ubiquitous and/or essential role of MED27 and Mediator complex throughout the CNS.

In conclusion, this case series provides a comprehensive clinical and radiological description of *MED27*-related disease, establishes genotype-phenotype and clinical-radiologic correlations and suggests differential diagnoses with other cerebello-lental degeneration syndromes and ‘neuro-MEDopathies’. Given the evidence of the progression of cerebellar features on neuroimaging, our series suggests an underlying neurodegenerative process that prompts us to define cerebellar involvement as ‘atrophy’ rather than ‘hypoplasia’ in *MED27*-related NDDs. Finally, the report proposes the term ‘MEDopathies’ for all the monogenic disorders resulting from defects in different subunits of the MED complex because of their commonalities in aetiology, pathology and clinical presentation. Further research is needed to improve our understanding of the pathobiology of *MED27*-related disease.

## Supplementary Material

awad257_Supplementary_Data

## Data Availability

The authors declare that the data supporting the findings of this study are available within the paper and its Supplementary material.

## References

[1] Malik S , Roeder RG. The metazoan mediator co-activator complex as an integrative hub for transcriptional regulation. Nat Rev Genet. 2010;11:761–772. 20940737 10.1038/nrg2901 PMC3217725

[2] Allen BL , Taatjes DJ. The mediator complex: A central integrator of transcription. Nat Rev Mol Cell Biol. 2015;16:155–166. 25693131 10.1038/nrm3951 PMC4963239

[3] Kaufmann R , Straussberg R, Mandel H, et al Infantile cerebral and cerebellar atrophy is associated with a mutation in the MED17 subunit of the transcription preinitiation mediator complex. Am J Hum Genet. 2010;87:667–670. 20950787 10.1016/j.ajhg.2010.09.016 PMC2978946

[4] Vodopiutz J , Schmook MT, Konstantopoulou V, et al MED20 Mutation associated with infantile basal ganglia degeneration and brain atrophy. Eur J Pediatr. 2015;174:113–118. 25446406 10.1007/s00431-014-2463-7

[5] Hashemi-Gorji F , Fardaei M, Tabei SMB, Miryounesi M. Novel mutation in the *MED*23 gene for intellectual disability: A case report and literature review. Clin Case Rep. 2019;7:331–335.. 30847200 10.1002/ccr3.1942 PMC6389469

[6] Basel-Vanagaite L , Smirin-Yosef P, Essakow JL, et al Homozygous MED25 mutation implicated in eye-intellectual disability syndrome. Hum Genet. 2015;134:577–587. 25792360 10.1007/s00439-015-1541-x

[7] Cali E , Ling S, Rocca C, et al A homozygous MED11 C-terminal variant causes a lethal neurodegenerative disease. Genet Med. 2022;24:2194–2203. 36001086 10.1016/j.gim.2022.07.013 PMC10519206

[8] Nizon M , Laugel V, Flanigan KM, et al Variants in MED12L, encoding a subunit of the mediator kinase module, are responsible for intellectual disability associated with transcriptional defect. Genet Med. 2019;21:2713–2722. 31155615 10.1038/s41436-019-0557-3 PMC7243155

[9] Rogers AP , Friend K, Rawlings L, Barnett CP. A de novo missense variant in MED13 in a patient with global developmental delay, marked facial dysmorphism, macroglossia, short stature, and macrocephaly. Am J Med Genet A. 2021;185:2586–2592. 33931951 10.1002/ajmg.a.62238

[10] Carvalho LML , da Costa SS, Campagnari F, et al Two novel pathogenic variants in MED13L: One familial and one isolated case. J Intellect Disabil Res. 2021;65:1049–1057. 34713510 10.1111/jir.12891

[11] Calpena E , Hervieu A, Kaserer T, et al De Novo missense substitutions in the gene encoding CDK8, a regulator of the mediator Complex, cause a syndromic developmental disorder. Am J Hum Genet. 2019;104:709–720. 30905399 10.1016/j.ajhg.2019.02.006 PMC6451695

[12] Chung HL , Mao X, Wang H, et al De novo variants in CDK19 are associated with a syndrome involving intellectual disability and epileptic encephalopathy. Am J Hum Genet. 2020;106:717–725. 32330417 10.1016/j.ajhg.2020.04.001 PMC7212481

[13] Lyons MJ . MED12-related disorders. In: Adam MP, Ardinger HH, Pagon RA, et al eds. Genereviews^®^. University of Washington; 2008.

[14] El Khattabi L , Zhao H, Kalchschmidt J, et al A pliable mediator acts as a functional rather than an architectural bridge between promoters and enhancers. Cell. 2019;178:1145–1158.e20. 31402173 10.1016/j.cell.2019.07.011 PMC7533040

[15] Meng L , Isohanni P, Shao Y, et al MED27 Variants cause developmental delay, dystonia, and cerebellar hypoplasia. Ann Neurol. 2021;89:828–833. 33443317 10.1002/ana.26019

[16] Reid KM , Spaull R, Salian S, et al MED27, SLC6A7, and MPPE1 variants in a Complex neurodevelopmental disorder with severe dystonia. Mov Disord. 2022;37:2139–2146. 35876425 10.1002/mds.29147 PMC9796674

[17] Sobreira N , Schiettecatte F, Valle D, Hamosh A. Genematcher: A matching tool for connecting investigators with an interest in the same gene. Hum Mutat. 2015;36:928–930. 26220891 10.1002/humu.22844 PMC4833888

[18] Richards S , Aziz N, Bale S, et al Standards and guidelines for the interpretation of sequence variants: A joint consensus recommendation of the American college of medical genetics and genomics and the association for molecular pathology. Genet Med. 2015;17:405–424. 25741868 10.1038/gim.2015.30 PMC4544753

[19] Dusek P , Smolinski L, Redzia-Ogrodnik B, et al Semiquantitative scale for assessing brain MRI abnormalities in wilson disease: A validation study. Mov Disord. 2020;35:994–1001. 32181965 10.1002/mds.28018

[20] Chaudhury S , Lyskov S, Gray JJ. Pyrosetta: A script-based interface for implementing molecular modeling algorithms using Rosetta. Bioinformatics. 2010;26:689–691. 20061306 10.1093/bioinformatics/btq007 PMC2828115

[21] Wang RY , Song Y, Barad BA, Cheng Y, Fraser JS, DiMaio F. Automated structure refinement of macromolecular assemblies from cryo-EM maps using Rosetta. Elife. 2016;5:e17219. 27669148 10.7554/eLife.17219 PMC5115868

[22] Alford RF , Leaver-Fay A, Jeliazkov JR, et al The Rosetta all-atom energy function for macromolecular modeling and design. J Chem Theory Comput. 2017;13:3031–3048. 28430426 10.1021/acs.jctc.7b00125 PMC5717763

[23] Schenkelberg CD , Bystroff C. Protein backbone ensemble generation explores the local structural space of unseen natural homologs. Bioinformatics. 2016;32:1454–1461. 26787668 10.1093/bioinformatics/btw001 PMC5006151

[24] Ferla MP , Pagnamenta AT, Damerell D, Taylor JC, Marsden BD. Michelanglo: Sculpting protein views on web pages without coding. Bioinformatics. 2020;36:3268–3270. 32061125 10.1093/bioinformatics/btaa104 PMC7214029

[25] GTEx Consortium; Laboratory, Data Analysis & Coordinating Center (LDACC)-Analysis Working Group, Statistical Methods groups-Analysis Working Group, et al Genetic effects on gene expression across human tissues. Nature. 2017;550:204–213. 29022597 10.1038/nature24277 PMC5776756

[26] Lupski JR . Clan genomics: From OMIM phenotypic traits to genes and biology. Am J Med Genet A. 2021;185:3294–3313. 34405553 10.1002/ajmg.a.62434 PMC8530976

[27] Poretti A , Boltshauser E. Terminology in morphological anomalies of the cerebellum does matter. Cerebellum Ataxias. 2015;2:8. 26331051 10.1186/s40673-015-0027-x PMC4552363

[28] Rüsch CT , Bölsterli BK, Kottke R, Steinfeld R, Boltshauser E. Pontocerebellar hypoplasia: A pattern recognition approach. Cerebellum. 2020;19:569–582. 32410094 10.1007/s12311-020-01135-5

[29] Smol T , Petit F, Piton A, et al MED13L-related Intellectual disability: Involvement of missense variants and delineation of the phenotype. Neurogenetics. 2018;19:93–103. 29511999 10.1007/s10048-018-0541-0

[30] Tørring PM , Larsen MJ, Brasch-Andersen C, et al Is MED13L-related intellectual disability a recognizable syndrome? Eur J Med Genet. 2019;62:129–136. 29959045 10.1016/j.ejmg.2018.06.014

[31] Haynes D , Pollack L, Prasad C, et al Further delineation of Basel-Vanagaite-Smirin-Yosef syndrome: Report of three patients. Am J Med Genet A. 2020;182:1785–1790. 32324310 10.1002/ajmg.a.61603

[32] Schor NF , Bianchi DW. Neurodevelopmental clues to neurodegeneration. Pediatr Neurol. 2021;123:67–76. 34399111 10.1016/j.pediatrneurol.2021.07.012 PMC10040214

[33] Deneubourg C , Ramm M, Smith LJ, et al The spectrum of neurodevelopmental, neuromuscular and neurodegenerative disorders due to defective autophagy. Autophagy. 2022;18:496–517. 34130600 10.1080/15548627.2021.1943177 PMC9037555

[34] Papandreou A , Danti FR, Spaull R, Leuzzi V, Mctague A, Kurian MA. The expanding spectrum of movement disorders in genetic epilepsies. Dev Med Child Neurol. 2020;62:178–191. 31784983 10.1111/dmcn.14407

