# Supplementary material for: Biallelic MED27 variants lead to variable ponto-cerebello-lental degeneration with movement disorders
Source: Brain. 2023 Jul 30;146(12):5031–43. doi: 10.1093/brain/awad257 (PMC10690011; doi:10.1093/brain/awad257)

Supplementary Material for "Biallelic *MED27* pathogenic variants lead to variable ponto-cerebello-lental degeneration with movement disorders"

Supplementary Materials and Methods

Supplementary Methods 1. Neuroimaging review

Supplementary Methods 2. Multiple sequence alignment

Supplementary Videos (doi: 10.6084/m9.figshare.23723940)

Supplementary Video 1

Supplementary Video 2

Supplementary Video 3

Supplementary Video 4

Supplementary Video 5

Supplementary Video 6

Supplementary Video 7

Supplementary Video 8

Supplementary Video 9

Supplementary Figures

Supplementary Figure 1. Haplotype and conservation analysis

Supplementary Figure 2. Phenotype severity

Supplementary Figure 3. Basal ganglia anomalies in subjects with *MED27*-related disease

Supplementary Figure 4. Cerebellar atrophy in subjects with *MED27*-related disease

Supplementary Figure 5. Evolution of neuroimaging findings in 12 subjects with *MED27* variants

Supplementary Figure 6. Leigh-like neuroimaging features in 2 subjects with *MED27* variants

Supplementary Tables

Supplementary Table 1. *MED27* variant characteristics and genetic methods

Supplementary Table 2. Detailed clinical features of the cases with biallelic *MED27* variants

Supplementary Table 3. Neuroimaging features and clinical-neuroradiological associations in MED27-related disease

Supplementary Table 4. Evolution of the neuroimaging features in 12 subjects with follow-up brain MRI studies

Supplementary Table 5. Differences in median age at last MRI according to neuroimaging features

Supplementary Table 6. Differential diagnosis of *MED27* with other MEDopathies

Supplementary Table 7. Differential diagnosis for *MED27*-related disease

Supplementary Results

Supplementary Discussion

Supplementary Literature

Supplementary Materials and Methods

Supplementary Methods 1. Neuroimaging review

Qualitative and semi-quantitative assessments of the cerebellar atrophy and supratentorial white matter volume reduction were performed as previously reported[1]. Additional information regarding the presence of cerebellar cortex T2 hyperintensity, cerebellar dentate nuclei T2 hyperintensity, pontine hypoplasia, degeneration of transverse pontocerebellar tracts and median pontine raphe nuclei (aka "hot cross bun sign"), and olivary nuclei degeneration were noticed. Moreover, basal ganglia were carefully assessed for the presence of atrophy and signal alterations. Finally, presence of white matter signal alterations, corpus callosum abnormalities, enlargement of the CSF spaces, simplified gyral pattern nodules of gray matter heterotopia, and olfactory bulb signal alterations were noted. Continuous variables were summarized as mean, and categorical variables were summarized as frequencies and percentages. Age differences at last brain MRI for clinical and neuroimaging features were tested using the Mann-Whitney U test. The associations between clinical and neuroradiological findings were

evaluated by the Chi-squared and Fisher exact test. Statistical significance was set at $p = 0.05$. Statistical analyses were performed using SPSS Statistics software, v26 (IBM, Armonk, NY, USA).

Supplementary Methods 2. Multiple sequence alignment

A multiple sequence alignment was constructed by aligning with Muscle[2] the MED27 protein sequence from the following species: human (Homo sapiens, Q6P2C8), mouse (Mus musculus, AAH16537.1), zebrafish (Danio rerio, NP_956954.1), fruitfly (Drosophila melanogaster, NP_649569.1), stony coral (Orbicella faveolate, XP_020601219.1), a basal fungus (Mortierella antarctica, KAF9985203.1), thalecress (Arabidopsis thaliana, NP_001325465.1). The inclusion of M. antarctica was driven by the loss of MED27 is the fungal crown group orders, which includes the ascomycotan Saccharomyces cerevisiae.

Supplementary Video Legends

Supplementary Video 1. This video shows the affected siblings from Family 3.

F3:P1 has perioral dyskinesia, tongue dystonia, elbow joint contractures, dystonia in the upper limbs, clenched fists, and hypertrophy of the sternocleidomastoid muscle on the left side (most likely due to dystonia).

F3:P2 has generalized dystonia with perioral dyskinesia, tongue and jaw dystonia, an opisthotonic extension of the trunk, and strabismus.

Supplementary Video 2. This video shows the affected sibling from Family 5.

Segments 1 and 2 show F5:P1who has generalized dystonia with perioral dyskinesia and tongue dystonia.

F5:P2 has dystonia in the upper limbs and perioral dyskinesia together with jaw-opening dystonia.

Supplementary Video 3. This video shows the affected sibling from Family 11.

F11:P1 shows a mild gait imbalance and difficulty performing tandem gait.

F11:P2 shows an unremarkable gait.

Supplementary Video 4. This video shows the affected siblings from Family 24.

F24:P1 has a jerky rest tremor in her right hand, a suggestion of tongue dystonia, dysarthric speech, a suggestion of jaw dystonia, and bilateral rest tremor in the upper limbs. The right-hand tremor worsens upon extending her arm suggesting a clonic-myoclonic tremor component, and her gait is clumsy.

F24:P2 shows a suggestion of right leg dystonia and slightly dysarthric speech.

F24:P3 has an inverted left foot upon walking

F24:P4 has generalized dystonia involving the posturing of the upper limbs, stooping of his body, dystonic tremor in his hands, lower limb dystonia, impaired speech with dysarthria, and perioral dyskinesia.

F24:P5 drags her left leg, probably due to dystonia and her gait is unstable. Her speech is dysarthric.

F24:P6 walks with feet slightly inverted and has a rest tremor in her right hand.

F24:P7 has a sardonic smile, forward bending of the trunk, right>left inverted feet on walking, mild flexion of her right knee joint, and dystonic flexion of her left arm.

F24:P8 has very mild perioral dyskinesia.

F24:P9 has an abnormal head and vertical eye movements suggestive of oculomotor apraxia or oculogyric upgaze.

Supplementary Video 5. This video shows the affected siblings from Family 25.

F25:P1 has antero-laterocolis with perioral dyskinesia and tongue dystonia. His right fist is clenched and there is generalized muscle weakness.

F25:P1 has no head control and stiffness in the limbs.

Supplementary Video 6. This video shows the affected individual from Family 26.

Segment 1 shows F26:P1 who is unable to stand up and has hypotonia.

Segment 2 shows F26:P1 has an unstable gait and drooling.

Segment 3 shows F26:P1 has an unsteady gait, mild hand dyskinesia, and head tremor.

Supplementary Video 7. This video shows the proband from Family 28.

Segment 1 shows the proband with generalized dystonia involving craniocervical muscles, upper and lower limbs, and his trunk with the episodes of opisthotonos movements. He has torticollis to the right with predominant hypertrophy of the left sternocleidomastoid muscle. He is not able to speak, has dysarthria, swallowing is impaired, and is unable to walk due to joint contractures and probable spastic-dystonia in the lower limbs. A periodic left myoclonic jerk in the left arm can be seen.

Segment 2 shows the proband with bilateral foot dystonia, toe deformities, knee joint contractures, and dry skin around the knees. Dystonia in the hands, neck, and perioral muscles could also be noticed.

Supplementary Video 8

Shows the proband from Family 29.

Segment 1 shows tongue and hand dystonia

Segment 2 shows a suggestion of axial hypotonia, tongue and hand dystonia, and contructures at the knee joints.

Segment 3 shows intermittent horizontal nystagmus, tongue dystonia, bilateral jerky tremor in the hands, and hand dystonia.

Supplementary Video 9

Shows the proband from Family 30.

Segment 1 shows mild gait incoordination and posturing of the left hand

Segment 2 shows mild hand dysmetria, left hand dystonia and mild positional jerky hand tremor

Segment 3 shows slightly dysarthric speech

Segment 4 shows hand dystonia and tremor

Supplementary Figures Legends

Supplementary Figure 1. Haplotype and conservation analysis. A) Pictorial representation of Exonic variants from 6 probands across ~22 Mb of chromosome 9q33.1-q34. 3 encompassing the homozygous missense *MED27* variants. Colour codes are as follows: homozygous single nucleotide polymorphisms (SNPs) as blue, heterozygous SNPs as yellow, and wild type as grey. Probands carrying homozygous p.His179Pro variant (green bar) showed similar haplotypes pattern (highlighted in pink). Probands carrying homozygous p.Gly291Ser (red bar) showed at least 2 distinct haplotype patterns surrounding the variant (Family 9 and 26, highlighted in green; Family 9, 26 and 24, highlighted in blue; Family 28, highlighted in orange). B) Multiple sequence alignment of diverse species showing the spans where pathogenic variants have been found (red squares). The variants are colour coded based on whether in silico analyses from Supplementary Results predict the to be destabilising (red, ΔΔGibbs > +2 kcal/mol), affecting a phosphorylation site (blue) or reduce flexibility (green).

Supplementary Figure 2. Genotype-phenotype correlation in *MED27*-related disease.

Three levels of the phenotype severity are shown and the carriers of the same *MED27* variants are highlighted in red, green, blue, yellow and brown.

Supplementary Figure 3. Basal ganglia anomalies in subjects with *MED27*-related disease. Axial T2-weighted or FLAIR images demonstrate variable degree of volume reduction of the caudate nuclei and/or putamina with additional diffuse caudate and putaminal signal alterations (thick arrows) or more focal hyperintensities of the posterior portions of the putamina in the majority of patients (arrowheads). Note the different degree of periventricular white matter volume reduction with ventricular enlargement and cortical atrophy in some affected subjects.

Supplementary Figure 4. Cerebellar atrophy in subjects with *MED27*-related disease. Sagittal T1 or T2-weighted images reveal mild to severe cerebellar atrophy with prevalent superior cerebellar vermis involvement in all affected subjects (thick arrows). Note the associated pontine hypoplasia (arrowheads) and thin corpus callosum (dashed arrows).

Supplementary Figure 5. Evolution of neuroimaging findings in 12 subjects with *MED27* variants. There is mild to moderate progression of the cerebellar atrophy in all subjects either at the level of the cerebellar vermis (thick arrows) and cerebellar hemispheres (thin arrows). Some of the patients develop also atrophy with signal alterations of the basal ganglia (arrowheads) and loss of white matter volume with thinning of the corpus callosum (empty arrows).

Supplementary Figure 6. Leigh-like neuroimaging features in 2 subjects with *MED27* variants. A-C) Axial T2-weighted and D-F) diffusion weighted images in F9:P2 reveal swelling and T2 hyperintensity with restricted diffusion at the level of the inferior olivary nuceli (thick arrows) associated with multiple symmetrical similar lesions at the level of the central midbrain (white arrowheads), superior colliculi (black arrowheads), cerebral

peduncles (dashed arrows) and subthalamic nuclei (empty arrows). G-I) Axial T2-weighted and J-L) diffusion weighted images in F26:P1 show symmetrical focal hyperintensities in the pontine tegmentum in the region of the medial longitudinal fasciculus (thin arrows) and lateral spinothalamic tracts (dashed arrows), in the central midbrain (arrowheads) and subthalamic nuclei (empty arrows).

Supplementary Tables

Supplementary Table 1. *MED27* variant characteristics and genetic methods. *Available as a separate excel table*

Supplementary Table 2. Detailed clinical features of the cases with biallelic *MED27* variants. *Available as a separate excel table*

Supplementary Table 3. Neuroimaging features and clinical-neuroradiological associations in *MED27*-related disease

	**N° Subjects (%)**	**Severe clinical phenotype (N=21)**	**P**	**Not ambulant (N= 14/34)**	**P**	**Ataxia (N= 12/20)**	**P**	**Dystonia/Dyskinesia (N=24/34)**	**P**
**PCF features**									
Cerebellar atrophy	34/34 (100)*								
moderate/severe CA	13/34 (38.2)	13/21 (61.9)	**<.001**	10/14 (71.4)	**.001**	2/12 (16.7)	1	11/24 (45.8)	.358
Cerebellar cortex T2 hyperintensity	6/34 (17.6)	6/21 (28.6)	.062	6/14 (42.9)	**.004**	0/12 (0)	NA	6/24 (25)	.302
Cerebellar dentate nuclei T2 hyperintensity	7/34 (20.5)	6/21 (28.6)	.210	6/14 (42.9)	**.028**	0/12 (0)	.200	6/24 (25)	1
Pontine hypoplasia	16/34 (47)	12/21 (57.1)	.172	8/14 (57.1)	.479	4/12 (33.3)	.1	12/24 (50)	.196
Hot cross bun sign°	3/34 (8.8)	3/21 (14.3)	.270	3/14 (21.4)	.081	0/12 (0)	NA	3/24 (12.5)	1
ON degeneration	3/34 (8.8)	3/21 (14.3)	1	2/14 (14.3)	.196	0/12 (0)	NA	2/24 (8.3)	1
**Basal ganglia anomalies**									
BG atrophy and signal alterations	15/34 (44.1)	11/21 (52.4)	.296	9/14 (64.3)	.076	3/12 (25)	1	12/24 (50)	.657
Severe CN volume reduction	14/34 (32.3)	10/19 (52.6)	.700	8/13 (61.5)	.257	2/10 (20)	.091	10/22 (45.5)	1
Severe putamen volume reduction	16/34 (44.1)	12/24 (66.7)	1	9/13 (69.2)	.685	3/6 (50)	.464	12/19 (63.2)	1
CN signal alterations	11/34 (32.3)	6/21 (28.6)	.709	5/14 (35.7)	1	4/12 (33.3)	1	8/24 (33.3)	1
Putamen signal alterations	15/34 (44.1)	11/21 (52.4)	.296	9/14 (64.3)	.076	3/12 (25)	1	12/24 (50)	.657
**Other supratentorial anomalies**									
WM volume reduction/ ventricular enlargement	26/34 (76.4)	20/21 (95.2)	**.002**	14/14 (100)	**.009**	5/12 (41.7)	.200	21/24 (87.5)	**.016**

Moderate/severe WM volume reduction	13/34 (38.2)	13/20 (65)	**.015**	10/14 (71.4)	**.036**	1/5 (20)	1	11/21 (52.4)	.478
WM signal alterations	19/34 (55.8)	14/21 (66.7)	.160	12/14 (85.7)	**.024**	4/12 (33.3)	.525	15/24 (62.5)	.660
Thin corpus callosum	12/34 (35.2)	11/21 (52.4)	**.011**	9/14 (64.3)	**.007**	0/12 (0)	.200	11/24 (45.8)	.061
Enlarged CSF spaces	24/34 (70.5)	17/21 (81.4)	.130	13/14 (92.9)	**.045**	4/12 (33.3)	.077	19/24 (79.2)	**.049**
PVNH	2/34 (5.8)	1/20 (5)	1	1/13 (7.7)	1	1/10 (10)	1	2/21 (9.5)	1
OB signal alterations	5/34 (14.7)	4/20 (20)	.640	3/13 (23.1)	.648	1/9 (11.1)	.455	4/22 (18.2)	1

Legend: BG, basal ganglia; CA, cerebellar atrophy; CN, caudate nuclei; CSF, cerebrospinal fluid; OB, olfactory bulbs; ON, Olivary nuclei; PVNH, periventricular nodular heterotopia; WM, white matter

*All subjects had a variable degree of cerebellar atrophy, ranging from very mild to severe

° The hot cross bun sign refers to the MRI appearance of the pons when T2 hyperintensity forms a cross on axial images, representing selective degeneration of transverse pontocerebellar tracts and median pontine raphe nuclei

Supplementary Table 4. Evolution of the neuroimaging features in 12 subjects with follow-up brain MRI studies

	Age first MRI, yrs	Age second MRI, yrs	FU duration, yrs	MRI findings
**F8.P1**	1.9	3	1.2	Mild progression of CA, WM volume loss, enlargement CSF spaces, no BG changes
**F14.P1**	1.8	3	1.3	Progression of CA, WM volume loss, enlargement of CSF spaces, BG atrophy and signal alterations (post putamen +++), OB signal alterations
**F15.P1**	0.5	1.7	1.2	Progression of CA, WM volume loss, enlargement of CSF spaces, loss of BG volume without signal alterations
**F19.P1**	0.9	2	1.1	Mild progression of CA, mild BG volume loss
**F20.P1**	10	11	1	Progression of CA, WM volume loss, enlargement of CSF spaces, BG atrophy and signal alterations
**F20.P2**	1	2.5	1.5	Progression of CA, WM volume loss, enlargement of CSF spaces, BG atrophy and signal alterations (post putamen +++)
**F22.P3**	1.5	8	6.5	Mild progression of CA
**F23.P1**	3	18	15	Mild progression of CA
**F25.P1**	7	9	2	Mild progression of CA, WM volume loss, increased signal alterations of the posterior putamen
**F25.P2**	1.1	4	3	Mild progression of CA, WM volume loss
**F27.P1**	1	10	9	Mild progression of CA, marked WM volume loss, enlargement of CSF spaces, BG atrophy and signal alterations
**F29.P1**	1	2	1	Severe progression of CA, marked WM volume loss and enlargement of CSF spaces, BG atrophy and signal alterations

Legend: BG, basal ganglia; CA, cerebellar atrophy; CSF, cerebrospinal fluid; OB, olfactory bulbs; yrs, years; WM, white matter.

Supplementary Table 5. Differences in median age at last MRI according to neuroimaging features

	Median Age at last MRI, years (IQR)	P
**Degree of cerebellar atrophy**		
Very mild - Mild	5 (6.2)	.780
Moderate - Severe	7 (8.6)	
**Cerebellar cortex T2-SA**		
No	5.5 (7.3)	.947
yes	5.7 (7.2)	
**Cerebellar dentate nuclei T2-SA**		
No	6 (7)	.677
Yes	4 (6.5)	
**Pontine hypoplasia**		
No	5 (8.3)	.484
Yes	6 (7.9)	
**Hot cross bun sign**		
No	6 (6.5)	.564
Yes	2.5 (NA)	
**Olivary nuclei degeneration**		
No	6 (6.5)	.645
Yes	2.5 (NA)	
**Basal ganglia atrophy and T2-SA**		
No	4 (6)	**.033**
Yes	9 (12)	
**Caudate nuclei VL**		
No	4.7 (13.1)	.897
Yes	5.5 (6.7)	
**Degree caudate VL**		
Normal-Mild	4 (4)	**.033**
Moderate-Severe	9 (12.8)	
**Putamen VL**		.985

No	4.5 (6.1)	
Yes	6.5 (8.6)	
**Degree putamen VL**		
Normal-Mild	2.7 (5.6)	.136
Moderate-Severe	7.7 (11.3)	
**Caudate nuclei T2-SA**		
No	3 (6)	**.001**
Yes	11 (13)	
**Putamen T2-SA**		
No	4 (6)	**.033**
Yes	9 (12)	
**White matter VL**		
No	8 (13.7)	.070
Yes	4 (7)	
**Degree white matter VL**		
Mild	3 (6.5)	.840
Moderate-Severe	7 (7.4)	
**White matter T2-SA**		
No	5 (12)	.681
Yes	7 (6.6)	
**Thin CC**		
No	4.5 (6.625)	.488
Yes	3 (10)	
**Enlarged CSF spaces**		
No	6.5 (7.5)	.423
Yes	4.5 (7.6)	
**Olfactory bulb T2-SA**		
No	4 (7)	.552
Yes	6 (8.2)	

Legend: CC, corpus callosum; IQR, inter-quartile range; NA, not applicable; PNH, periventricular nodular heterotopias; T2-SA, T2 signal alterations; VL, volume loss

Supplementary Table 6. Differential diagnosis of *MED27* with other MEDopathies. *Available as a separate excel table.*

Supplementary Table 7. Differential diagnosis for *MED27*-related disorder. *Available as a separate excel table.*

Supplementary Results

In silico modelling

MED27 is composed of two regions, a N-terminal region that forms a heterodimeric helical bundle with MED29 and the other a C-terminal globular domain that interacts with various head segment proteins (Fig. 2C and 2D). Except for one variant allele, the translated products of the variants affect residues within the globular domain. The only variant that affects the MED27-MED29 heterodimeric region is p.Val63Gly which is a structurally destabilising substitution, affecting a residue close to the interface with MED29 (Fig. 2D). The majority of the variants in the globular domain are predicted to be destabilising in the conformation analysed ($\Delta\Delta G > +2$ kcal/mol). The variant p.Pro259Leu is predicted to be strongly stabilising (–6.9 kcal/mol) and rigidifying (42% of the RMSD of a brief ensemble). The variant p.Ser232Phe is predicted to be neutral in terms of stability (+0.5 kcal/mol), as a result in a trade in side chain interactions. The nearby variants p.Ile230Arg and p.Val242Ala are predicted to be destabilising (+2.3 kcal/mol, +2.1 kcal/mol), but less so than the other variants. The inframe deletion p.Ser74_Val77del removes four residues in the structured loop between the first and second helix bundle with MED29 (Fig. 2D). The lack of these residues alters the position of the N-terminal helix most likely preventing bundle formation with MED29. In PhosphoSitePlus[3] the C-terminal residues Ser177, Ser181, Tyr222, Ser232, Ser234, Tyr236

and Thr242 are found phosphorylated in high-throughput studies, the latter five cluster together in a region where p.Ile230Arg, p.Ser232Phe, and p.Val242Ala reside (Fig. 2D).

The variant predicted to be the most destabilising to the conformation is p.Gly291Ser (>+10 kcal/mol), which is in a buried hairpin and causes several backbone and sidechain alterations. None of the C-terminal variants affect the strength of the interaction with the head protein. As a reference for variants that are potentially neutral, gnomAD, a database of variants from the healthy population, was inspected. There are only three stop-gain/frameshift and 42 missense variants reported in the canonical transcript (Q6P2C8, ENST00000292035, NM_004269, 311 AA long), which is the sole transcript in the tissues reported in the GTex database[4]. Only one variant, p.Val203Ile, is homozygous and in only one individual; this rare variant (0.00012) is predicted to be destabilising (+3.7 kcal/mol) in part due to the weakening of the interaction with MED17 (+1.3 kcal/mol), but given that the substitution is from a hydrophobic residue to a slightly larger one this could be an overestimate. Another rare variant (0.00012), p.Pro174Ala, is in the C-terminal domain and is predicted to be destabilising (+3 kcal/mol). p.Ser26Phe (0.000061) mapping to the N-terminus is predicted to be destabilising (+2 kcal/mol). The remaining variants are either structurally neutral or occur in amino acids within the regions of missing density.

Supplementary Discussion

With an accurate structure of only one conformation, it is not possible to predict which is more likely. Nevertheless, the picture may be more complex. The variant p.(Val203Ile) from gnomAD may help shed light on the mechanism, but the variant may not actually be destabilising in vivo to any conformation or the individual with the variant may not be healthy or they may not homozygous for that variant. Additionally, some variants in this cohort were predicted to be near a cluster of phosphorylated residues found in high throughput studies[3]: this

may be either coincidental or indicative of an unreported regulatory mechanism. The mediator complex triggers the phosphorylation of the RNA polymerase II as a result of a conformational change[5], therefore it is possible that some variants may favour one conformation over the other. Two overlapping hypotheses are possible for the mechanism that gives rise to a pathogenic phenotype in MED27: reduced mediator conformational switching or reduced functionally active complexes. A less stable protein variant might partake in mediator complex formation, but result in a lowered mediator activity, presumably due to hampered conformational switching, or it might be less likely to participate in mediator complex formation due to its aggregation and degradation, effectively lowering the concentration of functional active complexes. Understanding the phenotypes associated with MEDopathies can additionally help in understanding those involved in transcription factors and vice versa. Transcription factors generally possess a structured DNA-binding N-terminal domain, frequently dimeric, and a C-terminal tail, that is repeat-rich, disordered in isolation and frequently found modified post-translationally in high-throughput studies, which is involved in recruiting the mediator complex, by wrapping itself across particular regions of the complex[6]. With the advances endowed by AlphaFold2 in modelling protein-protein interactions[7], it may be possible to iteratively across subsection of the mediator to determine the precise binding location of the C-terminal tail of a transcription factor and better understand how the transcript initialisation signals are integrated and the subtle differences between different MEDopathies.

Supplementary literature

1. Dusek P, Smolinski L, Redzia-Ogrodnik B, et al. Semiquantitative Scale for Assessing Brain MRI Abnormalities in Wilson Disease: A Validation Study. Mov Disord. 2020 Jun;35(6):994-1001. doi: 10.1002/mds.28018.
2. Robert C. Edgar, MUSCLE: multiple sequence alignment with high accuracy and high throughput, Nucleic Acids Research, Volume 32, Issue 5, 1 March 2004, Pages 1792–1797, https://doi.org/10.1093/nar/gkh340

3. Peter V. Hornbeck, Bin Zhang, et al., PhosphoSitePlus, 2014: mutations, PTMs and recalibrations, Nucleic Acids Research, Volume 43, Issue D1, 28 January 2015, Pages D512–D520, https://doi.org/10.1093/nar/gku1267
4. GTEx Consortium; Laboratory, Data Analysis &Coordinating Center (LDACC)—Analysis Working Group; Statistical Methods groups—Analysis Working Group; Genetic effects on gene expression across human tissues [published correction appears in Nature. 2017 Dec 20;:]. *Nature*. 2017;550(7675):204-213. doi:10.1038/nature24277
5. El Khattabi L, Zhao H, Kalchschmidt J, et al. A Pliable Mediator Acts as a Functional Rather Than an Architectural Bridge between Promoters and Enhancers. *Cell*. 2019;178(5):1145-1158.e20. doi:10.1016/j.cell.2019.07.011
6. Meyer KD, Lin SC, Bernecky C, Gao Y, Taatjes DJ. p53 activates transcription by directing structural shifts in Mediator. Nat Struct Mol Biol. 2010 Jun;17(6):753-60. doi: 10.1038/nsmb.1816.
7. Jumper J, Evans R, Pritzel A, et al. Highly accurate protein structure prediction with AlphaFold. Nature. 2021 Aug;596(7873):583-589. doi: 10.1038/s41586-021-03819-2.

Supplementary Table 6. Differential diagnosis of *MED27* with other MEDopathies

1. Hashimoto S, Boissel S, Zarhrate M, et al. MED23 mutation links intellectual disability to dysregulation of immediate early gene expression. *Science*. 2011;333(6046):1161-1163. doi:10.1126/science.1206638
2. Goh YS, Grants JM. Mutations in the Mediator subunit MED23 link intellectual disability to immediate early gene regulation. *Clin Genet*. 2012;81(5):430. doi:10.1111/j.1399-0004.2011.01821.x
3. Trehan A, Brady JM, Maduro V, et al. MED23-associated intellectual disability in a non-consanguineous family. *Am J Med Genet A*. 2015;167(6):1374-1380. doi:10.1002/ajmg.a.37047
4. Lionel AC, Monfared N, Scherer SW, Marshall CR, Mercimek-Mahmutoglu S. MED23-associated refractory epilepsy successfully treated with the ketogenic diet. *Am J Med Genet A*. 2016;170(9):2421-2425. doi:10.1002/ajmg.a.37802
5. Hashemi-Gorji F, Fardaei M, Tabei SMB, Miryounesi M. Novel mutation in the *MED23* gene for intellectual disability: A case report and literature review. *Clin Case Rep*. 2019;7(2):331-335. Published 2019 Jan 9. doi:10.1002/ccr3.1942
6. Ignatius E, Isohanni P, Pohjanpelto M, et al. Genetic background of ataxia in children younger than 5 years in Finland. *Neurol Genet*. 2020;6(4):e444. Published 2020 Jun 5. doi:10.1212/NXG.0000000000000444
7. Basel-Vanagaite L, Smirin-Yosef P, Essakow JL, et al. Homozygous MED25 mutation implicated in eye-intellectual disability syndrome. *Hum Genet*. 2015;134(6):577-587. doi:10.1007/s00439-015-1541-x
8. Figueiredo T, Melo US, Pessoa AL, et al. Homozygous missense mutation in MED25 segregates with syndromic intellectual disability in a large consanguineous family. *J Med Genet*. 2015;52(2):123-127. doi:10.1136/jmedgenet-2014-102793
9. Nair P, Sabbagh S, Bizzari S, et al. Report of a Second Lebanese Family with Basel-Vanagaite-Smirin-Yosef Syndrome: Possible Founder Mutation. *Mol Syndromol*. 2019;10(4):219-222. doi:10.1159/000501114

10. Haynes D, Pollack L, Prasad C, et al. Further delineation of Basel-Vanagaite-Smirin-Yosef syndrome: Report of three patients. *Am J Med Genet A*. 2020;182(7):1785-1790. doi:10.1002/ajmg.a.61603
11. Maini I, Errichiello E, Caraffi SG, et al. Improving the phenotype description of Basel-Vanagaite-Smirin-Yosef syndrome, MED25-related: polymicrogyria as a distinctive neuroradiological finding. *Neurogenetics*. 2021;22(1):19-25. doi:10.1007/s10048-020-00625-2
12. Kaufmann R, Straussberg R, Mandel H, et al. Infantile cerebral and cerebellar atrophy is associated with a mutation in the MED17 subunit of the transcription preinitiation mediator complex. *Am J Hum Genet*. 2010;87(5):667-670. doi:10.1016/j.ajhg.2010.09.016
13. Hirabayashi S, Saitsu H, Matsumoto N. Distinct but milder phenotypes with choreiform movements in siblings with compound heterozygous mutations in the transcription preinitiation mediator complex subunit 17 (MED17). *Brain Dev*. 2016;38(1):118-123. doi:10.1016/j.braindev.2015.05.004
14. Agostini A, Marchetti D, Izzi C, et al. Expanding the phenotype of MED 17 mutations: Description of two new cases and review of the literature. *Am J Med Genet B Neuropsychiatr Genet*. 2018;177(8):687-690. doi:10.1002/ajmg.b.32677
15. Fattal-Valevski A, Ben Sira L, Lerman-Sagie T, et al. Delineation of the phenotype of MED17-related disease in Caucasus-Jewish families. *Eur J Paediatr Neurol*. 2021;32:40-45. doi:10.1016/j.ejpn.2020.08.011
16. Vodopiutz J, Schmook MT, Konstantopoulou V, et al. MED20 mutation associated with infantile basal ganglia degeneration and brain atrophy. *Eur J Pediatr*. 2015;174(1):113-118. doi:10.1007/s00431-014-2463-7
17. Calpena E, Hervieu A, Kaserer T, et al. De Novo Missense Substitutions in the Gene Encoding CDK8, a Regulator of the Mediator Complex, Cause a Syndromic Developmental Disorder. *Am J Hum Genet*. 2019;104(4):709-720. doi:10.1016/j.ajhg.2019.02.006
18. Uehara T, Abe K, Oginuma M, et al. Pathogenesis of CDK8-associated disorder: two patients with novel CDK8 variants and in vitro and in vivo functional analyses of the variants. *Sci Rep*. 2020;10(1):17575. Published 2020 Oct 16. doi:10.1038/s41598-020-74642-4
19. Sugawara Y, Mizuno T, Moriyama K, et al. Cerebrospinal fluid abnormalities in developmental and epileptic encephalopathy with a *de novo CDK19* variant. *Neurol Genet*. 2020;6(6):e527. Published 2020 Oct 8. doi:10.1212/NXG.0000000000000527
20. Yang S, Yu W, Chen Q, Wang X. A novel variant of *CDK19* causes a severe neurodevelopmental disorder with infantile spasms. *Cold Spring Harb Mol Case Stud*. 2021;7(2):a006082. Published 2021 Apr 8. doi:10.1101/mcs.a006082
21. Zarate YA, Uehara T, Abe K, et al. CDK19-related disorder results from both loss-of-function and gain-of-function de novo missense variants. *Genet Med*. 2021;23(6):1050-1057. doi:10.1038/s41436-020-01091-9
22. Chung HL, Mao X, Wang H, et al. De Novo Variants in CDK19 Are Associated with a Syndrome Involving Intellectual Disability and Epileptic Encephalopathy. *Am J Hum Genet*. 2020;106(5):717-725. doi:10.1016/j.ajhg.2020.04.001
23. Rubinato E, Rondeau S, Giuliano F, et al. MED12 missense mutation in a three-generation family. Clinical characterization of MED12-related disorders and literature review. *Eur J Med Genet*. 2020;63(3):103768. doi:10.1016/j.ejmg.2019.103768
24. Polla DL, Bhoj EJ, Verheij JBGM, et al. De novo variants in MED12 cause X-linked syndromic neurodevelopmental disorders in 18 females. *Genet Med*. 2021;23(4):645-652. doi:10.1038/s41436-020-01040-6
25. Gonzalez A, Kapur S, Walsh M, Vengoechea J. Two females with distinct de novo missense pathogenic variants in MED12 and vastly differing phenotypes. *Am J Med Genet A*. 2021;185(8):2582-2585. doi:10.1002/ajmg.a.62233

26. Shah A, Bapna M, Al-Saif H, Li R, Couser NL. Eye and ocular adnexa manifestations of *MED12*-related disorders [published online ahead of print, 2021 Oct 20]. *Ophthalmic Genet*. 2021;1-4. doi:10.1080/13816810.2021.1989601
27. Nizon M, Laugel V, Flanigan KM, et al. Variants in MED12L, encoding a subunit of the mediator kinase module, are responsible for intellectual disability associated with transcriptional defect [published correction appears in Genet Med. 2019 Jul 3;:]. *Genet Med*. 2019;21(12):2713-2722. doi:10.1038/s41436-019-0557-3
28. Rogers AP, Friend K, Rawlings L, Barnett CP. A de novo missense variant in MED13 in a patient with global developmental delay, marked facial dysmorphism, macroglossia, short stature, and macrocephaly. *Am J Med Genet A*. 2021;185(8):2586-2592. doi:10.1002/ajmg.a.62238
29. Asadollahi R, Zweier M, Gogoll L, et al. Genotype-phenotype evaluation of MED13L defects in the light of a novel truncating and a recurrent missense mutation. *Eur J Med Genet*. 2017;60(9):451-464. doi:10.1016/j.ejmg.2017.06.004
30. Smol T, Petit F, Piton A, et al. MED13L-related intellectual disability: involvement of missense variants and delineation of the phenotype. *Neurogenetics*. 2018;19(2):93-103. doi:10.1007/s10048-018-0541-0
31. Dawidziuk M, Kutkowska-Kaźmierczak A, Gawliński P, et al. The *MED13L* haploinsufficiency syndrome associated with de novo nonsense variant (P.GLN1981*). *J Mother Child*. 2021;24(3):32-36. Published 2021 Apr 30. doi:10.34763/jmotherandchild.20202403.2021.d-20-00003
32. Carvalho LML, da Costa SS, Campagnari F, et al. Two novel pathogenic variants in MED13L: one familial and one isolated case. *J Intellect Disabil Res*. 2021;65(12):1049-1057. doi:10.1111/jir.12891
33. Bessenyei B, Balogh I, Mokánszki A, Ujfalusi A, Pfundt R, Szakszon K. *MED13L*-related intellectual disability due to paternal germinal mosaicism. *Cold Spring Harb Mol Case Stud*. 2022;8(1):a006124. Published 2022 Jan 10. doi:10.1101/mcs.a006124

Supplementary Table 7. Differential Diagnosis for *MED27*-related disorder

1. Gatti M, Magri S, Di Bella D, et al. Spastic paraplegia type 46: novel and recurrent GBA2 gene variants in a compound heterozygous Italian patient with spastic ataxia phenotype. *Neurol Sci*. 2021;42(11):4741-4745. doi:10.1007/s10072-021-05463-0
2. Kloth K, Cozma C, Bester M, Gerloff C, Biskup S, Zittel S. Dystonia as initial presentation of compound heterozygous GBA2 mutations: Expanding the phenotypic spectrum of SPG46. *Eur J Med Genet*. 2020;63(9):103992. doi:10.1016/j.ejmg.2020.103992
3. Anttonen AK. Marinesco-Sjögren Syndrome. In: Adam MP, Ardinger HH, Pagon RA, et al., eds. *GeneReviews®*. Seattle (WA): University of Washington, Seattle; November 29, 2006.
4. Bayram N, Kaçar Bayram A, Daimagüler HS, et al. Genotype-phenotype correlations in ocular manifestations of Marinesco-Sjögren syndrome: Case report and literature review [published online ahead of print, 2021 Jun 2]. *Eur J Ophthalmol*. 2021;11206721211021291. doi:10.1177/11206721211021291
5. Appelhof B, Wagner M, Hoefele J, et al. Pontocerebellar hypoplasia due to bi-allelic variants in MINPP1. *Eur J Hum Genet*. 2021;29(3):411-421. doi:10.1038/s41431-020-00749-x
6. Ucuncu E, Rajamani K, Wilson MSC, et al. MINPP1 prevents intracellular accumulation of the chelator inositol hexakisphosphate and is mutated in Pontocerebellar Hypoplasia. *Nat Commun*. 2020;11(1):6087. Published 2020 Nov 30. doi:10.1038/s41467-020-19919-y
7. Doi H, Ushiyama M, Baba T, et al. Late-onset spastic ataxia phenotype in a patient with a homozygous DDHD2 mutation. *Sci Rep*. 2014;4:7132. Published 2014 Nov 24. doi:10.1038/srep07132

8. Schuurs-Hoeijmakers JH, Geraghty MT, Kamsteeg EJ, et al. Mutations in DDHD2, encoding an intracellular phospholipase A(1), cause a recessive form of complex hereditary spastic paraplegia. *Am J Hum Genet*. 2012;91(6):1073-1081. doi:10.1016/j.ajhg.2012.10.017
9. Gonzalez M, Nampoothiri S, Kornblum C, et al. Mutations in phospholipase DDHD2 cause autosomal recessive hereditary spastic paraplegia (SPG54). *Eur J Hum Genet*. 2013;21(11):1214-1218. doi:10.1038/ejhg.2013.29
10. Novarino G, Fenstermaker AG, Zaki MS, et al. Exome sequencing links corticospinal motor neuron disease to common neurodegenerative disorders. *Science*. 2014;343(6170):506-511. doi:10.1126/science.1247363
11. Erfanian Omidvar M, Torkamandi S, Rezaei S, et al. Genotype-phenotype associations in hereditary spastic paraplegia: a systematic review and meta-analysis on 13,570 patients. *J Neurol*. 2021;268(6):2065-2082. doi:10.1007/s00415-019-09633-1
12. Arnoldi A, Tonelli A, Crippa F, et al. A clinical, genetic, and biochemical characterization of SPG7 mutations in a large cohort of patients with hereditary spastic paraplegia. *Hum Mutat*. 2008;29(4):522-531. doi:10.1002/humu.20682
13. Elleuch N, Depienne C, Benomar A, et al. Mutation analysis of the paraplegin gene (SPG7) in patients with hereditary spastic paraplegia. *Neurology*. 2006;66(5):654-659. doi:10.1212/01.wnl.0000201185.91110.15
14. Sánchez-Ferrero E, Coto E, Beetz C, et al. SPG7 mutational screening in spastic paraplegia patients supports a dominant effect for some mutations and a pathogenic role for p.A510V. *Clin Genet*. 2013;83(3):257-262. doi:10.1111/j.1399-0004.2012.01896.x
15. Kara E, Tucci A, Manzoni C, et al. Genetic and phenotypic characterization of complex hereditary spastic paraplegia. *Brain*. 2016;139(Pt 7):1904-1918. doi:10.1093/brain/aww111
16. Abdel-Hamid MS, Abdel-Ghafar SF, Ismail SR, et al. Micro and Martsolf syndromes in 34 new patients: Refining the phenotypic spectrum and further molecular insights. *Clin Genet*. 2020;98(5):445-456. doi:10.1111/cge.13825
17. Handley MT, Morris-Rosendahl DJ, Brown S, et al. Mutation spectrum in RAB3GAP1, RAB3GAP2, and RAB18 and genotype-phenotype correlations in warburg micro syndrome and Martsolf syndrome. *Hum Mutat*. 2013;34(5):686-696. doi:10.1002/humu.22296
18. Handley M, Sheridan E. RAB18 Deficiency. In: Adam MP, Ardinger HH, Pagon RA, et al., eds. *GeneReviews®*. Seattle (WA): University of Washington, Seattle; January 4, 2018.
19. Le Van Quyen P, Calmels N, Bonnière M, et al. Prenatal diagnosis of cerebro-oculo-facio-skeletal syndrome: Report of three fetuses and review of the literature. *Am J Med Genet A*. 2020;182(5):1236-1242. doi:10.1002/ajmg.a.61520
20. Jaspers NG, Raams A, Silengo MC, et al. First reported patient with human ERCC1 deficiency has cerebro-oculo-facio-skeletal syndrome with a mild defect in nucleotide excision repair and severe developmental failure. *Am J Hum Genet*. 2007;80(3):457-466. doi:10.1086/512486
21. Drury S, Boustred C, Tekman M, et al. A novel homozygous ERCC5 truncating mutation in a family with prenatal arthrogryposis--further evidence of genotype-phenotype correlation. *Am J Med Genet A*. 2014;164A(7):1777-1783. doi:10.1002/ajmg.a.36506
22. Laugel V, Dalloz C, Tobias ES, et al. Cerebro-oculo-facio-skeletal syndrome: three additional cases with CSB mutations, new diagnostic criteria and an approach to investigation. *J Med Genet*. 2008;45(9):564-571. doi:10.1136/jmg.2007.057141
23. Reunert J, van den Heuvel A, Rust S, Marquardt T. Cerebro-oculo-facio-skeletal syndrome caused by the homozygous pathogenic variant Gly47Arg in ERCC2. *Am J Med Genet A*. 2021;185(3):930-936. doi:10.1002/ajmg.a.62048
24. Haugarvoll K, Johansson S, Rodriguez CE, et al. GBA2 Mutations Cause a Marinesco-Sjögren-Like Syndrome: Genetic and Biochemical Studies. *PLoS One*. 2017;12(1):e0169309. Published 2017 Jan 4. doi:10.1371/journal.pone.0169309

25. Nie S, Chen G, Cao X, Zhang Y. Cerebrotendinous xanthomatosis: a comprehensive review of pathogenesis, clinical manifestations, diagnosis, and management. *Orphanet J Rare Dis*. 2014;9:179. Published 2014 Nov 26. doi:10.1186/s13023-014-0179-4
26. Stelten BML, van de Warrenburg BPC, Wevers RA, Verrips A. Movement disorders in cerebrotendinous xanthomatosis. *Parkinsonism Relat Disord*. 2019;58:12-16. doi:10.1016/j.parkreldis.2018.07.006
27. Wong JC, Walsh K, Hayden D, Eichler FS. Natural history of neurological abnormalities in cerebrotendinous xanthomatosis. *J Inherit Metab Dis*. 2018;41(4):647-656. doi:10.1007/s10545-018-0152-9
28. Ma C, Ren YD, Wang JC, et al. The clinical and imaging features of cerebrotendinous xanthomatosis: A case report and review of the literature. *Medicine (Baltimore)*. 2021;100(9):e24687. doi:10.1097/MD.0000000000024687
29. Gürbüz BB, Yılmaz DY, Coşkun T, Tokatlı A, Dursun A, Sivri HS. Glutaric aciduria type 1: Genetic and phenotypic spectrum in 53 patients. *Eur J Med Genet*. 2020;63(11):104032. doi:10.1016/j.ejmg.2020.104032
30. Wang Q, Li X, Ding Y, Liu Y, Song J, Yang Y. Clinical and mutational spectra of 23 Chinese patients with glutaric aciduria type 1. *Brain Dev*. 2014;36(9):813-822. doi:10.1016/j.braindev.2013.11.006
31. Kurkina MV, Mihaylova SV, Baydakova GV, et al. Molecular and biochemical study of glutaric aciduria type 1 in 49 Russian families: nine novel mutations in the GCDH gene. *Metab Brain Dis*. 2020;35(6):1009-1016. doi:10.1007/s11011-020-00554-x
32. Larson A, Goodman S. Glutaric Acidemia Type 1. In: Adam MP, Ardinger HH, Pagon RA, et al., eds. *GeneReviews®*. Seattle (WA): University of Washington, Seattle; September 19, 2019.
33. Kafil-Hussain NA, Monavari A, Bowell R, Thornton P, Naughten E, O'Keefe M. Ocular findings in glutaric aciduria type 1. *J Pediatr Ophthalmol Strabismus*. 2000;37(5):289-293.
34. Kyllerman M, Skjeldal O, Christensen E, et al. Long-term follow-up, neurological outcome and survival rate in 28 Nordic patients with glutaric aciduria type 1. *Eur J Paediatr Neurol*. 2004;8(3):121-129. doi:10.1016/j.ejpn.2003.12.007
35. Fiskerstrand T, H'mida-Ben Brahim D, Johansson S, et al. Mutations in ABHD12 cause the neurodegenerative disease PHARC: An inborn error of endocannabinoid metabolism. *Am J Hum Genet*. 2010;87(3):410-417. doi:10.1016/j.ajhg.2010.08.002
36. Nishiguchi KM, Avila-Fernandez A, van Huet RA, et al. Exome sequencing extends the phenotypic spectrum for ABHD12 mutations: from syndromic to nonsyndromic retinal degeneration [published correction appears in Ophthalmology. 2017 Feb;124(2):273-274]. *Ophthalmology*. 2014;121(8):1620-1627. doi:10.1016/j.ophtha.2014.02.008
37. Thimm A, Rahal A, Schoen U, et al. Genotype-phenotype correlation in a novel ABHD12 mutation underlying PHARC syndrome. *J Peripher Nerv Syst*. 2020;25(2):112-116. doi:10.1111/jns.12367
38. Lerat J, Cintas P, Beauvais-Dzugan H, Magdelaine C, Sturtz F, Lia AS. A complex homozygous mutation in ABHD12 responsible for PHARC syndrome discovered with NGS and review of the literature. *J Peripher Nerv Syst*. 2017;22(2):77-84. doi:10.1111/jns.12216
39. Nguyen XT, Almushattat H, Strubbe I, et al. The Phenotypic Spectrum of Patients with PHARC Syndrome Due to Variants in *ABHD12*: An Ophthalmic Perspective. *Genes (Basel)*. 2021;12(9):1404. Published 2021 Sep 11. doi:10.3390/genes12091404
40. Cohen BH, Chinnery PF, Copeland WC. *POLG*-Related Disorders. In: Adam MP, Ardinger HH, Pagon RA, et al., eds. *GeneReviews®*. Seattle (WA): University of Washington, Seattle; March 16, 2010.
41. Rahman S, Copeland WC. POLG-related disorders and their neurological manifestations. *Nat Rev Neurol*. 2019;15(1):40-52. doi:10.1038/s41582-018-0101-0

42. Stumpf JD, Saneto RP, Copeland WC. Clinical and molecular features of POLG-related mitochondrial disease. *Cold Spring Harb Perspect Biol*. 2013;5(4):a011395. Published 2013 Apr 1. doi:10.1101/cshperspect.a011395
43. Klouwer FC, Berendse K, Ferdinandusse S, Wanders RJ, Engelen M, Poll-The BT. Zellweger spectrum disorders: clinical overview and management approach. *Orphanet J Rare Dis*. 2015;10:151. Published 2015 Dec 1. doi:10.1186/s13023-015-0368-9
44. Braverman NE, Raymond GV, Rizzo WB, et al. Peroxisome biogenesis disorders in the Zellweger spectrum: An overview of current diagnosis, clinical manifestations, and treatment guidelines. *Mol Genet Metab*. 2016;117(3):313-321. doi:10.1016/j.ymgme.2015.12.009
45. Steinberg SJ, Raymond GV, Braverman NE, Moser AB. Zellweger Spectrum Disorder. In: Adam MP, Ardinger HH, Pagon RA, et al., eds. *GeneReviews®*. Seattle (WA): University of Washington, Seattle; December 12, 2003.
46. Chen YJ, Wang MW, Dong EL, et al. Chinese patients with adrenoleukodystrophy and Zellweger spectrum disorder presenting with hereditary spastic paraplegia. *Parkinsonism Relat Disord*. 2019;65:256-260. doi:10.1016/j.parkreldis.2019.06.008
47. Mignarri A, Vinciguerra C, Giorgio A, et al. Zellweger Spectrum Disorder with Mild Phenotype Caused by PEX2 Gene Mutations. *JIMD Rep*. 2012;6:43-46. doi:10.1007/8904_2011_102
48. Kashgari A. Neonate with classic Zellweger syndrome [published correction appears in Int J Pediatr Adolesc Med. 2020 Dec;7(4):213]. *Int J Pediatr Adolesc Med*. 2019;6(4):165-166. doi:10.1016/j.ijpam.2019.11.002
49. Mohebbi MR, Rush ET, Rizzo WB, Banagale RC. Zellweger syndrome and associated brain malformations: report of a novel Peroxin1 (PEX1) mutation in a Native American infant. *J Child Neurol*. 2012;27(12):1589-1592. doi:10.1177/0883073811435918
50. Berendse K, Engelen M, Ferdinandusse S, et al. Zellweger spectrum disorders: clinical manifestations in patients surviving into adulthood. *J Inherit Metab Dis*. 2016;39(1):93-106. doi:10.1007/s10545-015-9880-2
51. Lax NZ, Gnanapavan S, Dowson SJ, et al. Early-onset cataracts, spastic paraparesis, and ataxia caused by a novel mitochondrial tRNAGlu (MT-TE) gene mutation causing severe complex I deficiency: a clinical, molecular, and neuropathologic study. *J Neuropathol Exp Neurol*. 2013;72(2):164-175. doi:10.1097/NEN.0b013e31828129c5
52. Duan R, Shi Y, Yu L, et al. UBA5 Mutations Cause a New Form of Autosomal Recessive Cerebellar Ataxia. *PLoS One*. 2016;11(2):e0149039. Published 2016 Feb 12. doi:10.1371/journal.pone.0149039
53. Beaudin M, Klein CJ, Rouleau GA, Dupré N. Systematic review of autosomal recessive ataxias and proposal for a classification. *Cerebellum Ataxias*. 2017;4:3. Published 2017 Feb 23. doi:10.1186/s40673-017-0061-y
54. Scala M, De Grandis E, Nobile G, et al. Biallelic ZBTB11 variants associated with complex neuropsychiatric phenotype featuring Tourette syndrome. Brain. 2023;146(1):e1-e4. doi:10.1093/brain/awac323
55. Fattahi Z, Sheikh TI, Musante L, et al. Biallelic missense variants in ZBTB11 can cause intellectual disability in humans. Hum Mol Genet. 2018;27(18):3177-3188. doi:10.1093/hmg/ddy220
56. Sumathipala D, Strømme P, Fattahi Z, et al. ZBTB11 dysfunction: spectrum of brain abnormalities, biochemical signature and cellular consequences. Brain. 2022;145(7):2602-2616. doi:10.1093/brain/awac034
57. Ortigoza-Escobar et al (personal communication)

Thumbnail image references

1. Ophthalmic Atlas Images by EyeRounds.org, The University of Iowa are licensed under a Creative Commons Attribution-NonCommercial-NoDerivs 3.0 Unported License.
2. Fernández-Torre, José. (2010). Interictal EEG. Atlas of Epilepsies, First Edition; Published by Springer-Verlag. Editors: Panayioutopolous C

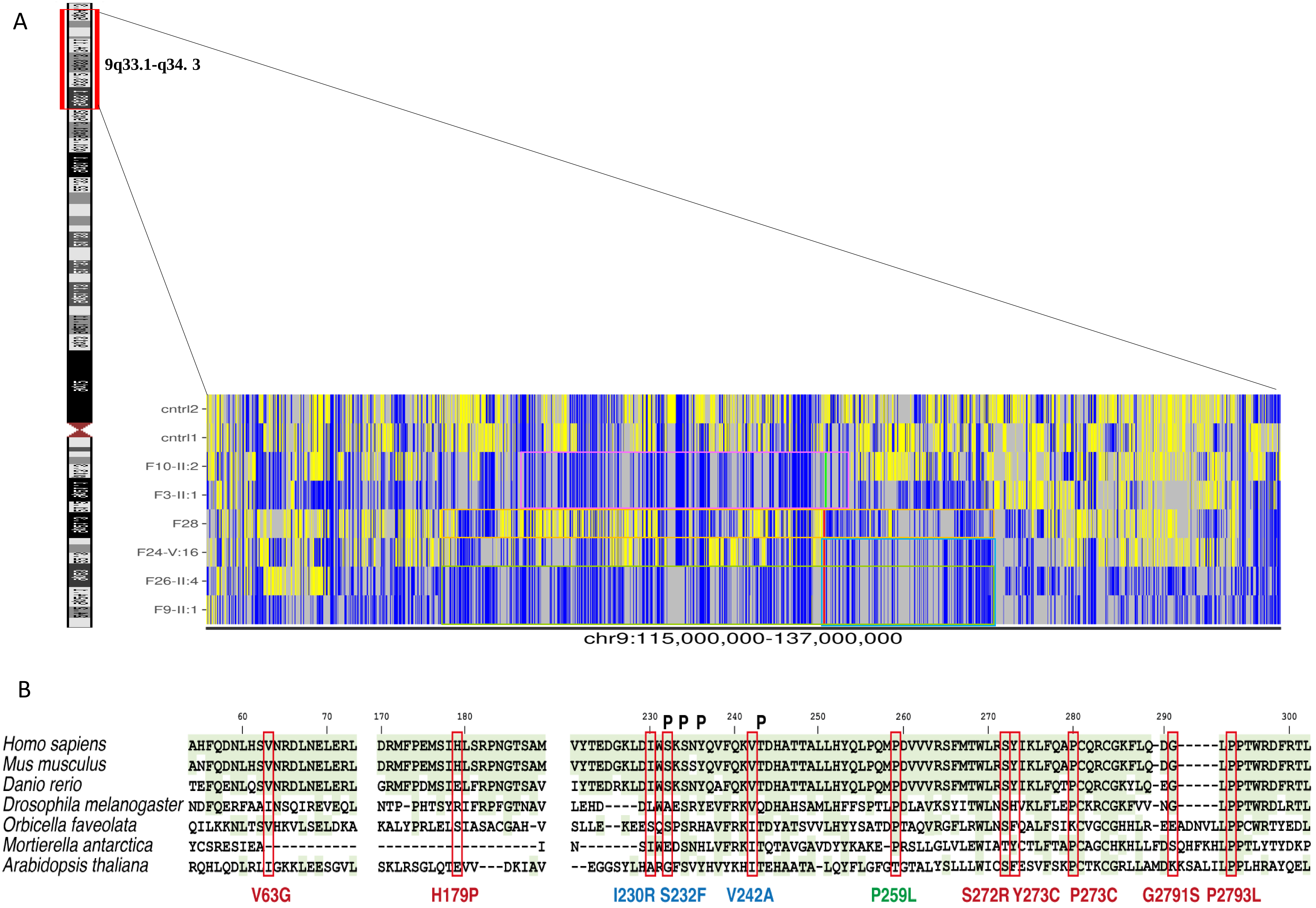

A
9q33.1-q34. 3
cntrl2
cntrl1
F10-II:2
F3-II:1
F28
F24-V:16
F26-II:4
F9-II:1
chr9:115,000,000-137,000,000
B
60
70
170
180
230
P P P
240
P
250
260
270
280
290
300
Homo sapiens AHFQDNLHSVNRDLNELERL DRMFPEMSIHLSRPNGTSAM VYTEDGKLDIWSKSNYQVFQKVTDHATTALLHYQLPQMPDVVVRSFMTWLRSYIKLFQAPCQRCGKFLQ-DG-----LPPTWRDFRTL
Mus musculus ANFQDNLHSVNRDLNELERL DRMFPEMSIHLSRPNGTSAM VYTEDGKLDIWSKSSYQVFQKVTDHATTALLHYQLPQMPDVVVRSFMTWLRSYIKLFQAPCQRCGKFLQ-DG-----LPPTWRDFRTL
Danio rerio TEFQENLQSVNRDLNELERL GRMFPDMSIELFRPNGTSAV IYTEDRKLDIWSKSNYQAFQKVTDHATTALLHYQLPQMPDVVVRSFMTWLRSYIKLFQTPCQRCGKYLQ-EG-----LPPTWRDFRTL
Drosophila melanogaster NDFQERFAAINSQIREVEQL NTP-PHTSYRIFRPFGTNAV LEHD----DLWAESRYEVFRKVQDHAHSAMLHFFSPTLPDLAVKSYITWLNSHVKLFLEPCKRCGKFVV-NG-----LPPTWRDLRTL
Orbicella faveolata QILKKNLTSVHKVLSELDKA KALYPRLELSIASACGAH-V SLLE--KEESQSPSRHAVFRKITDYATSVVLHYYSATDPTAQVRGFLRWLNSFQALFSIKCVGCGHHLR-EEADNVLLPPCWRTYEDL
Mortierella antarctica YCSRESIEA----------- -------------------I N-------SIWEDSNHLVFRKITQTAVGAVDYYKAKE-PRSLLGLVLEWIATYCTLFTAPCAGCHKHLLFDSQHFKHLPPTLYTYDKP
Arabidopsis thaliana RQHLQDLRLIGKKLEESGVL SKLRSGLQTEVV---DKIAV --EGGSYLHARGFSVYHVYKHITEHAATA-LQYFLGFGTGTALYSLLLWICSFESVFSKPCTKCGRLLAMDKKSALILPPLHRAYQEL
V63G
H179P
I230R S232F
V242A
P259L
S272R Y273C
P273C
G2791S P2793L

Supplementary Figure 2

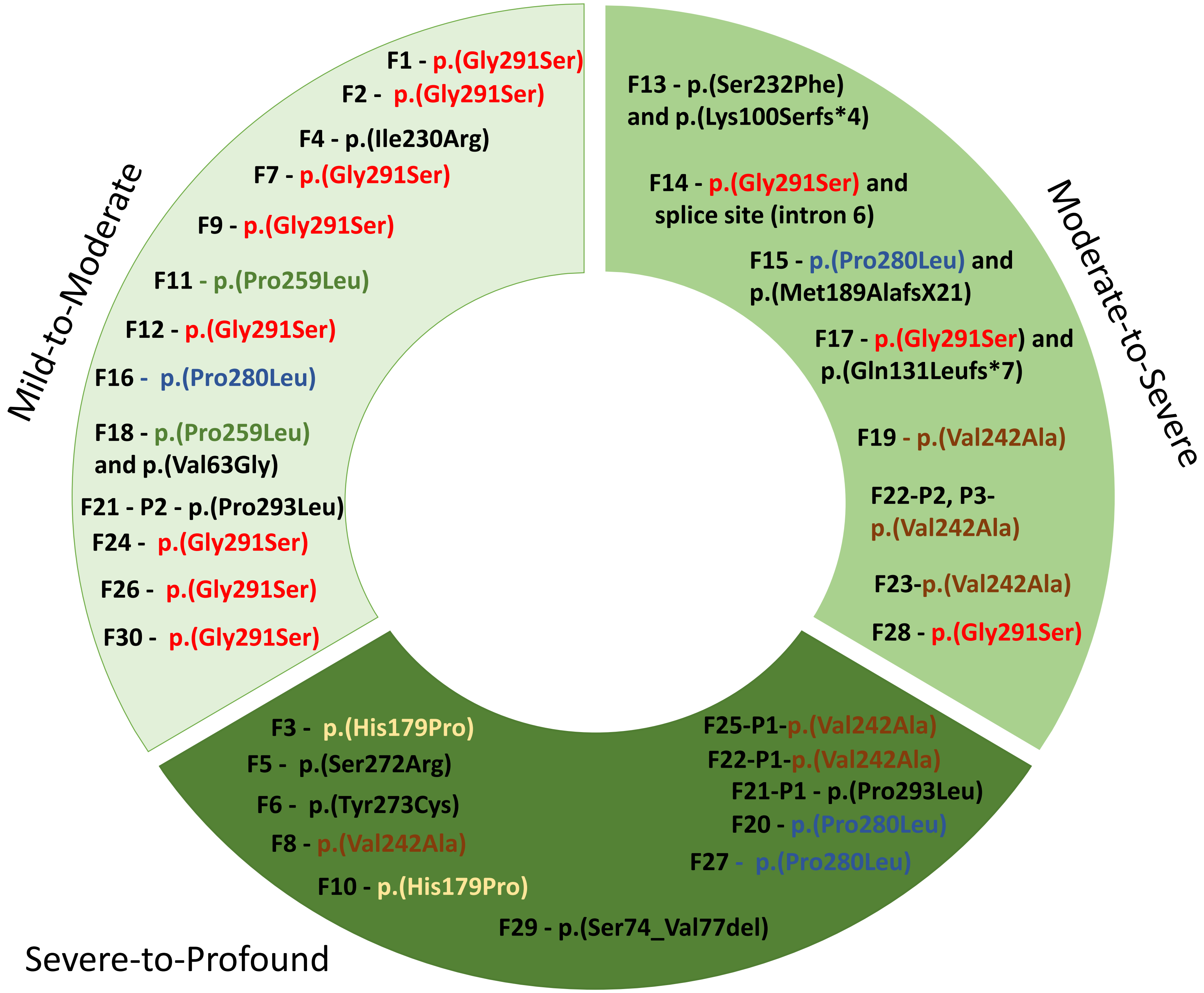

Supplementary Figure 3

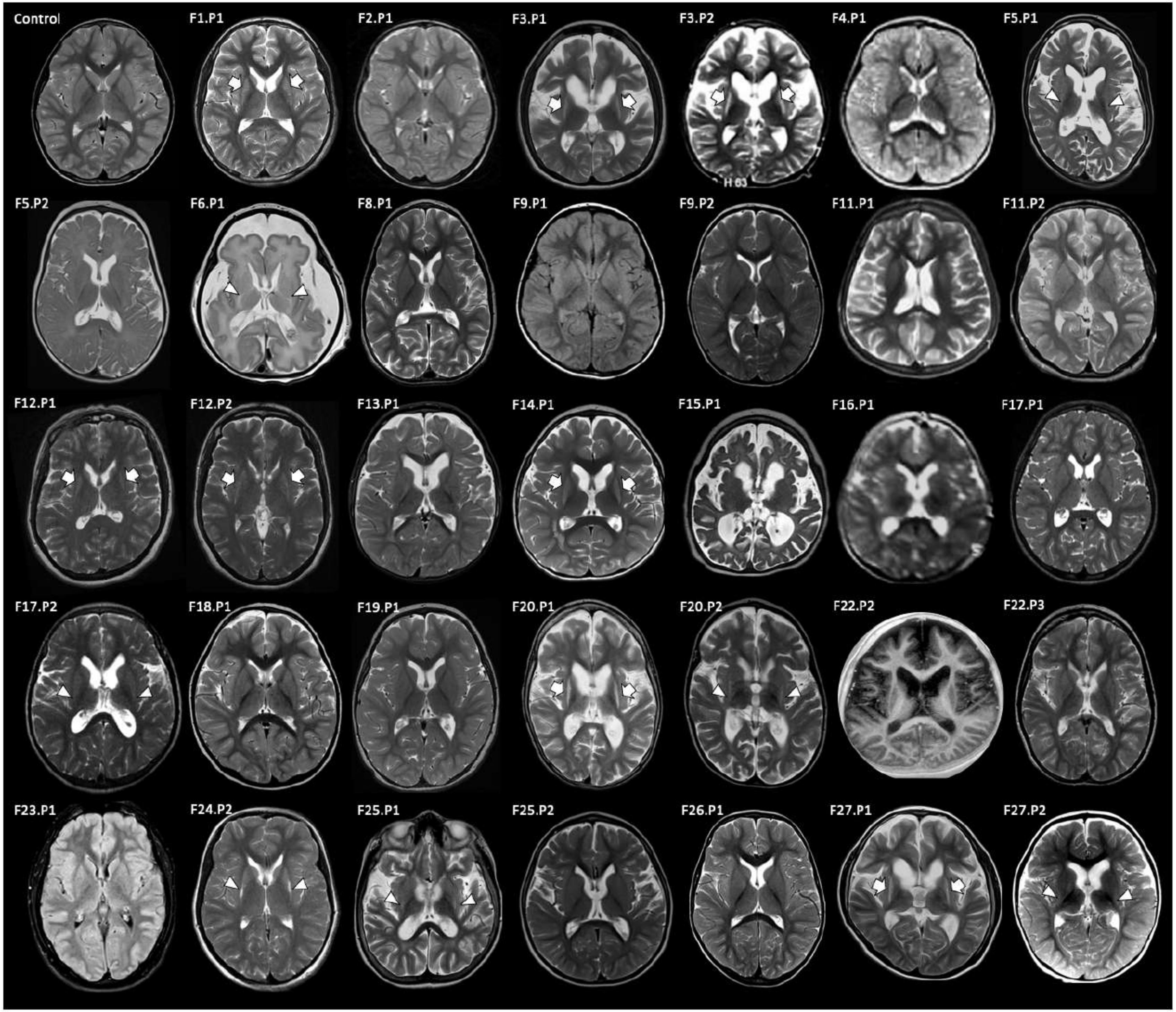

Supplementary Figure 4

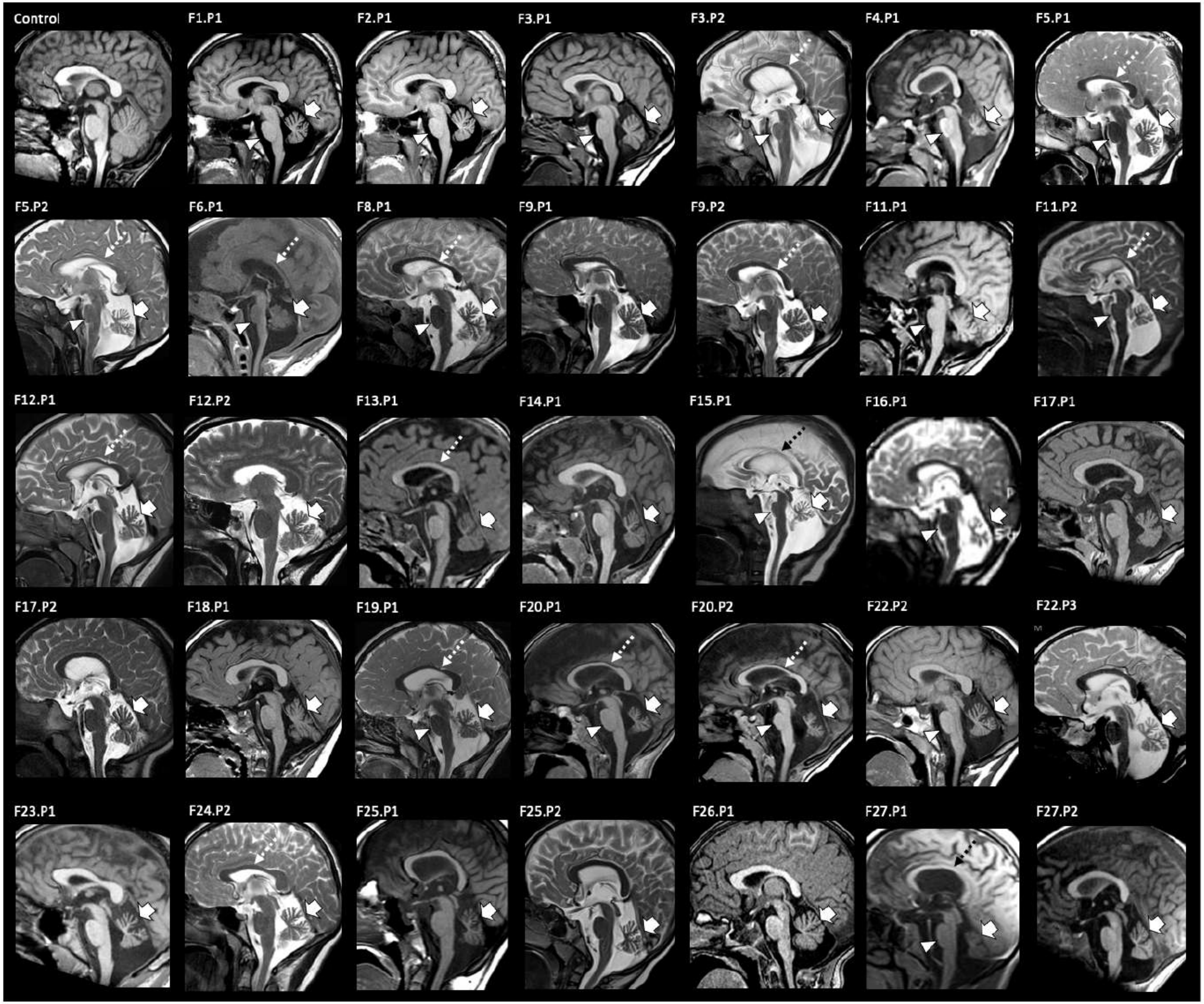

Supplementary Figure 5

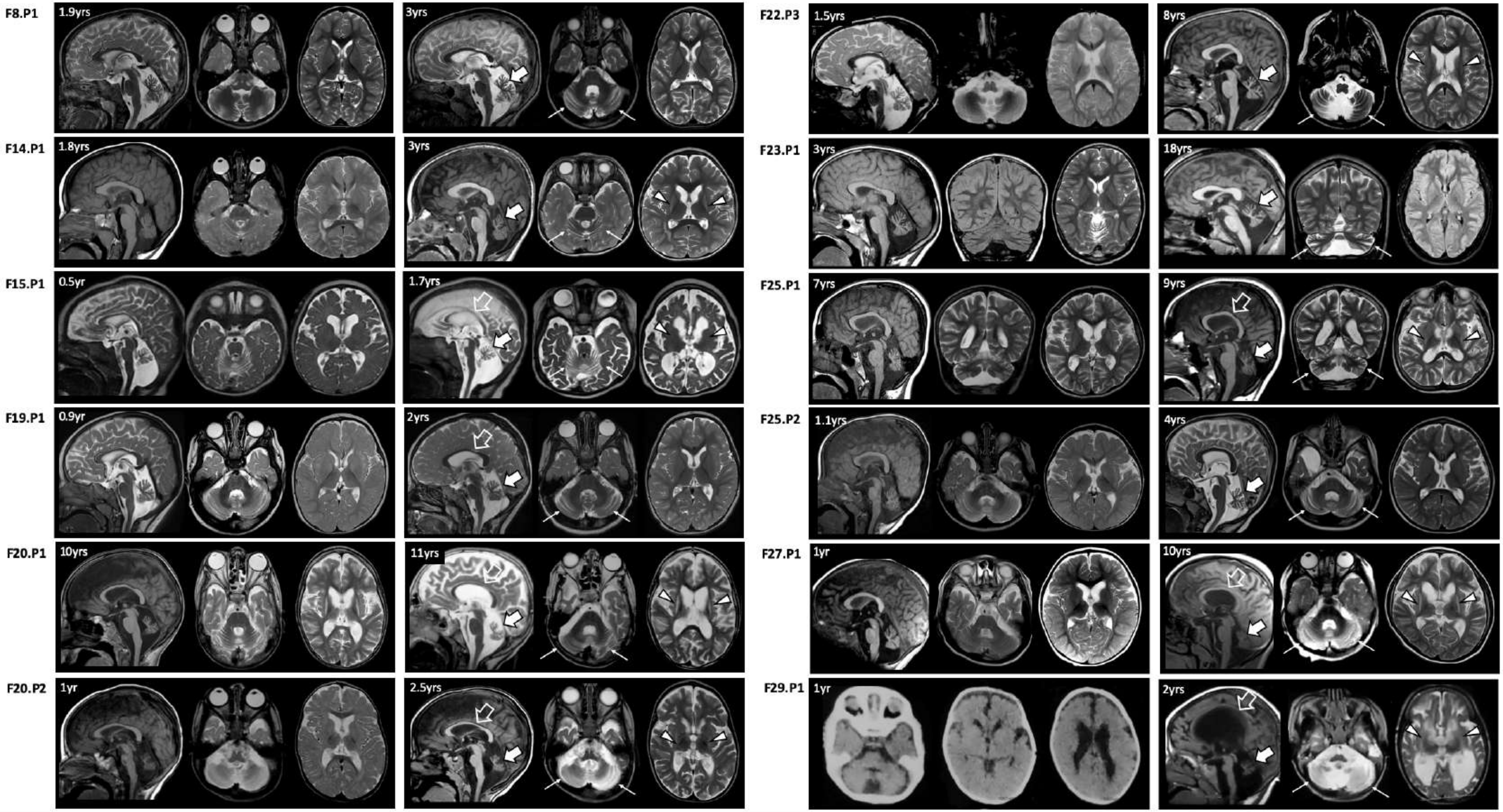

Supplementary Figure 6

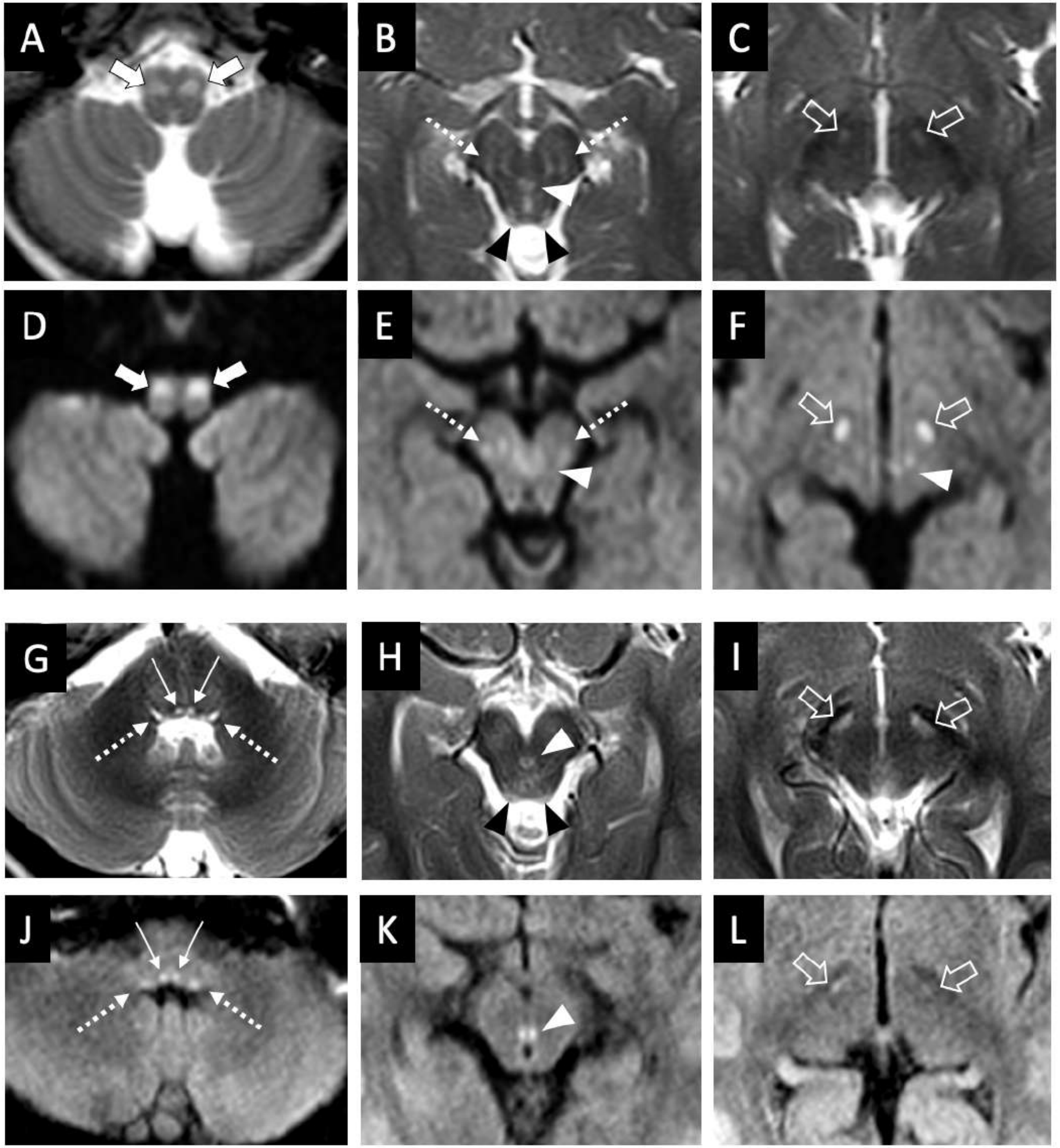

Supplement: awad257_Supplementary_Data [file awad257_supplementary_data.zip › brain-2023-00192-File007.pdf]
